# Noise Contributions in an Inducible Genetic Switch: A Whole-Cell Simulation Study

**DOI:** 10.1371/journal.pcbi.1002010

**Published:** 2011-03-10

**Authors:** Elijah Roberts, Andrew Magis, Julio O. Ortiz, Wolfgang Baumeister, Zaida Luthey-Schulten

**Affiliations:** 1 Department of Chemistry, University of Illinois at Urbana-Champaign, Urbana, Illinois, United States of America; 2 Center for the Physics of Living Cells, University of Illinois at Urbana-Champaign, Urbana, Illinois, United States of America; 3 Center for Biophysics and Computational Biology, University of Illinois at Urbana-Champaign, Urbana, Illinois, United States of America; 4 Department of Molecular Structural Biology, Max Planck Institute of Biochemistry, Martinsreid, Germany; Semmelweis University, Hungary

## Abstract

Stochastic expression of genes produces heterogeneity in clonal populations of bacteria under identical conditions. We analyze and compare the behavior of the inducible *lac* genetic switch using well-stirred and spatially resolved simulations for *Escherichia coli* cells modeled under fast and slow-growth conditions. Our new kinetic model describing the switching of the *lac* operon from one phenotype to the other incorporates parameters obtained from recently published *in vivo* single-molecule fluorescence experiments along with *in vitro* rate constants. For the well-stirred system, investigation of the intrinsic noise in the circuit as a function of the inducer concentration and in the presence/absence of the feedback mechanism reveals that the noise peaks near the switching threshold. Applying maximum likelihood estimation, we show that the analytic two-state model of gene expression can be used to extract stochastic rates from the simulation data. The simulations also provide mRNA–protein probability landscapes, which demonstrate that switching is the result of crossing both mRNA and protein thresholds. Using cryoelectron tomography of an *E. coli* cell and data from proteomics studies, we construct spatial *in vivo* models of cells and quantify the noise contributions and effects on repressor rebinding due to cell structure and crowding in the cytoplasm. Compared to systems without spatial heterogeneity, the model for the fast-growth cells predicts a slight decrease in the overall noise and an increase in the repressors rebinding rate due to anomalous subdiffusion. The tomograms for *E. coli* grown under slow-growth conditions identify the positions of the ribosomes and the condensed nucleoid. The smaller slow-growth cells have increased mRNA localization and a larger internal inducer concentration, leading to a significant decrease in the lifetime of the repressor–operator complex and an increase in the frequency of transcriptional bursts.

## Author Summary

Expressing genes in a bacterial cell is noisy and random. A colony of bacteria grown from a single cell can show remarkable differences in the copy number per cell of a given protein after only a few generations. In this work we use computer simulations to study the variation in how individual cells in a population express a set of genes in response to an environmental signal. The modeled system is the *lac* genetic switch that *Escherichia coli* uses to find, collect, and process lactose sugar from the environment. The noise inherent in the genetic circuit controlling the cell's response determines how similar the cells are to each other and we study how the different components of the circuit affect this noise. Furthermore, an estimated 30–50% of the cell volume is taken up by a wide variety of large biomolecules. To study the response of the circuit caused by crowding, we simulate the circuit inside a three-dimensional model of an *E. coli* cell built using data from cryoelectron tomography reconstructions of a single cell and proteomics data. Correctly including random effects of molecular crowding will be critical to developing fully dynamic models of living cells.

## Introduction

Transcriptional and translational regulatory networks control the phenotype of modern cells, regulating gene expression in response to changing environmental conditions and/or biological stimuli. It has been well established that intrinsic noise in gene regulation results from the discrete biochemical nature of the process [1]. There is also an extrinsic component to the total noise arising from cell-to-cell variation in the number of copies of the transcription and translation machinery (transcription factors, RNA polymerases, ribosomes, etc) [2]–[4]. Stochastic noise can lead to different phenotypic outcomes for a cellular population and, in certain fluctuating environments, the resulting heterogeneous population can be more optimal for growth than would be a population containing a single phenotype [5], [6].

Theoretical modeling of stochasticity in gene expression has been a topic of intense study in the last decade and has greatly increased our understanding of the effect that statistical noise has on gene regulation (for reviews see [7]–[11]). Without detailed information regarding spatial heterogeneity within a cell, models of stochastic gene expression are typically expressed in terms of the chemical master equation (CME), which describes the time evolution of the probability for a chemical system to be in a given state [12]. Various analytical methods including moment generating functions [1], [3], [13], the Langevin and Fokker-Planck equations [14], linear noise approximation [4], and many-body theory [15] are used to study such models of gene expression. Computer simulations, usually based on a variant of Gillespie's stochastic simulation algorithm (SSA) [16] are also widely employed to analyze gene network models that are too complex to be amenable to analytical modeling [17], [18].

Such theoretical studies have predicted and experimental measurements have shown [2], [19]–[23] that populations of cells can be quite heterogeneous, even when starting from an initially identical state. The large variance in the population distribution is usually ascribed to bursting in the process of gene transcription. Two models have been developed which can be used as a framework for quantitatively analyzing population distributions to infer the underlying gene expression kinetics.

The burst model (Figure 1A) of Friedman *et al.* [24] is based on the assumption that an mRNA's lifetime is short compared with that of its protein product. In that case, proteins will be produced in independent bursts with exponentially distributed sizes. The solution to the stationary probability distribution of protein 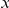 in the continuous CME formulation of the model was shown to be the Gamma distribution 
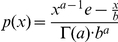
 where the 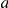 and 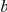 parameters were interpreted to be the frequency of transcriptional bursts relative to the protein lifetime and the mean number of proteins produced per burst, respectively.

**Figure 1:**
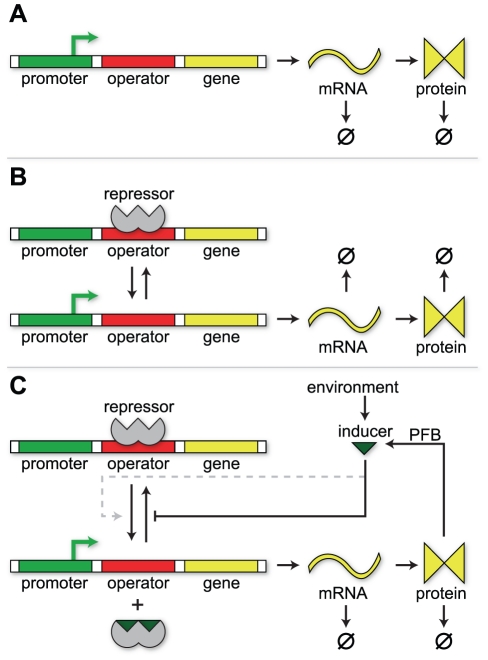
Three models for stochastic gene expression. (A) Burst model in which transcription of the DNA is always active. (B) Two-state model in which the DNA switches with constant rates between active and repressed states. (C) Inducible genetic switch in which an inducer both controls the rate of switching between active and inactive transcription states and is also positively regulated by the protein product – a positive feedback loop (PFB). The gray dotted connection indicates a weak effect of the inducer in promoting the unbinding of repressor at high inducer concentrations.

Shahrezaei and Swain [25] further developed the analytical theory of gene expression, by deriving not only the time-dependent probability distribution for the burst model, but also the steady-state distribution for a two-state model of gene expression (Figure 1B; three-stage model in their nomenclature). In the two-state model a gene alternates between transcriptionally active and inactive states with constant rates. Their analytical distributions show that in addition to large variance within a population, bimodality can appear when transitions between the active and inactive states are slow. A similar model has also been used to analyze the switching behavior of a population due to rare large events versus the cumulative effect of many small events [26].

Computational modeling can greatly assist in understanding genetic systems where complexity exceeds the capacity of analytical solutions. In a model for an inducible genetic switch incorporating more of the complexity present in real biological systems (Figure 1C), the transitions between the active and inactive transcriptional states are no longer constant but depend upon an external inducer likely in a nonlinear manner. The positive feedback (PFB) loop changes the network topology by introducing an additional regulatory link. Both of these differences provide additional sources of noise in the circuit that may affect the probability distributions. Combining computer modeling of a complete genetic circuit with analysis using simplified analytical models can help to provide an overall picture of the dynamics of such a system.

Further complexity in modeling real biological systems comes from the spatial heterogeneity within a cell and molecular crowding in the *in vivo* environment. It is becoming apparent that the cell is not a well-stirred system [27]–[29]. Studies using cryoelectron tomography techniques [30]–[34] have revealed that individual macromolecules are not necessarily uniformly distributed inside the cell, but may be clustered in a spatially dependent manner. Spatial organization can affect reaction kinetics by increasing local concentrations of reactants and enzymes. Additionally, crowding and non-specific molecular interactions in the *in vivo* environment can lead to anomalous subdiffusive behavior for macromolecules, as measured experimentally [35], [36] and by computational modeling of bacterial cytoplasmic environments [37]–[39]. Accounting for spatial heterogeneity is a challenge to computational biology that must eventually be met and several such modeling studies have been undertaken [37]–[44].

Stochastic modeling of gene expression circuits in a three-dimensional bacterial cell poses several difficulties, both computational and informational in nature. Recently a “lattice microbe” method [37] was developed using GPU (graphics processing unit) computational accelerators to simulate diffusion of macromolecules within a modeled *Escherichia coli* cell packed with a distribution of obstacles according to reported proteomics data. It implemented a multiparticle reaction-diffusion algorithm on a three-dimensional lattice to perform simulations of cell-scale systems. With the lattice microbe method one can observe anomalous diffusion of macromolecules and track diffusive-reactive processes over the timescale of the cell cycle, with spatial resolution from 2–16 nm. On the informational side, painstaking efforts must be undertaken to obtain parameters for the models. Kinetic parameters, which are often obtained under *in vitro* conditions, must be validated by comparing modeling results to published experiments. Recent time-lapse fluorescence microscopy experiments have been able to track dynamic behavior for individual macromolecules *in vivo* [21], [45], providing an additional source for model parameters. Parameters obtained from *in vivo* single-molecule experiments are uniquely suited for stochastic modeling, as they provide population distributions not simply mean values from ensemble measurements. Equally importantly such parameters are measured under *in vivo* conditions and incorporate the effects of the cellular environment. Also, super-resolution imaging studies [46]–[49] provide further spatial information to complement the cryoelectron tomography data.

We present here a computational study of gene expression noise in the inducible genetic switch shown in Figure 1C using both well-stirred and spatially resolved models. Spatial models of *E. coli* cells were constructed to approximate cytoplasmic crowding under both rapid and slow growth phenotypes, with the latter being based on data from cryoelectron tomography [50]. Both spatial models were simulated using the lattice microbe method [37]. The genetic switch was based on the well-characterized *E. coli* lactose utilization system, parameterized using measurements from a recent series of *in vivo* single-molecule fluorescence studies [21], [22], [51] as well as published *in vitro* rate constants. We report the contributions to intrinsic noise from the regulatory elements of the inducible genetic circuit as well as the extrinsic noise due to *in vivo* crowding. Using the slow-growth model we investigate the effect of using experimentally determined cellular architecture in reaction-diffusion models, with implications for effects due to cell growth. Comparing the noise from the inducible genetic switch to the bursting and two-state models described above (Figure 1A,B), we consider what improvements in both modeling and experimental efforts are needed to develop stochastic models of gene expression with predictive power regarding phenotype switching and heterogeneity in cellular populations.

## Methods

### Lac circuit kinetic model

#### Lactose uptake in *Escherichia coli*

**Figure 2:**
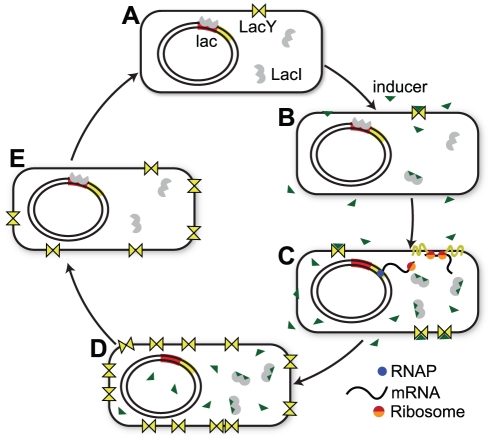
Overview of the *lac* genetic circuit in *E. coli*. (A) In the absence of inducer, the lac repressor (LacI) binds to the *lac* operator preventing transcription of genes in the *lac* operon. (B) Following an increase in the extracellular inducer concentration, inducer enters the cell via both diffusion across the membrane and active transport by lactose permease (LacY). Once inside, inducer binds free LacI molecules preventing them from binding to the operator. (C) After the intracellular inducer concentration reaches a threshold, any bound repressor is “knocked-off” the operator leading to expression of the *lac* genes. (D) At high intracellular inducer concentrations the genes for lactose metabolism are fully induced. (E) After inducer is removed, repressor rebinds to the operator preventing further expression of the *lac* operon and the enzymes for lactose metabolism are either degraded or diluted through cellular division.

The lactose utilization system in *E. coli* is a model system for studying inducible genetic circuits [52]–[58]. The overall genetic system is described in Figure 2. Briefly, the *lac* repressor (LacI; 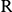 in model annotation) [59]–[61] binds to the *lac* operator (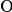 in model annotation) upstream of the DNA encoding for the genes responsible for lactose uptake and metabolism, repressing their expression in the absence of lactose. In the presence of lactose or another inducer (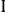 in model annotation), LacI binds the inducer preferentially and is prevented from binding to the operator region allowing expression of the proteins in the *lac* operon. One protein in the operon, lactose permease (LacY; 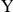 in model annotation), establishes positive feedback in the circuit by inserting into the membrane and actively transporting lactose into the cell, ensuring that LacI remains sequestered; the cell switches to the induced state. Theoretical and experimental studies have investigated the behavior of *lac* system and shown it to be stochastic, depending on random fluctuations to switch between the off and on states [62], [63]. We assumed the same overall kinetic structure for our model of the *lac* system as Stamatakis and Mantzaris [63], but where possible derived the rate parameters from single molecule *in vivo* experiments. The reactions and stochastic rate constants, derived using a well-stirred approximation, are given in Table 1.

**Table 1:** Reactions and rate constants used in the stochastic model of the *lac* circuit.

Reaction	Param	Stochastic Rate		Units	Source^a^		Pub. *in vitro* Rate
**Lac operon regulation**							
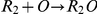	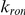	2.43e+06		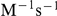	*M*		4.0-20.0e+08^b^
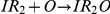	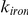	1.21e+06		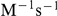	*M*		–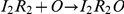	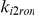	2.43e+04		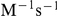	*M*		–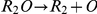	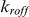	6.30e-04		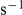	*S*		1.4-2.3e-02^b^
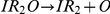	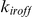	6.30e-04		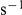	*S*		–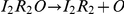	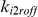	3.15e-01		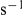	*M*		–
**Transcription, translation, and degredation**							
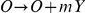	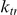	1.26e-01		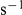	*M*		–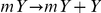	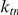	4.44e-02		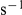	*S*		–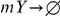	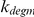	1.11e-02		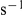	*S*		–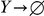	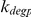	2.10e-04		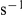	*M*		–
**Inducer–repressor interact.**		TMG	IPTG		TMG	IPTG	IPTG
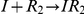	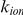	2.27e+04	9.71e+04	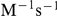	*M*	*K*	9.2-9.8e+04^c^
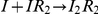	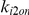	1.14e+04	4.85e+04	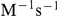	*M*	*K*	4.6-4.9e+04^c^
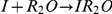	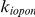	6.67e+02	2.24e+04	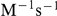	*M*	*K*	2.0-2.3e+04^c^
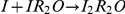	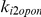	3.33e+02	1.12e+04	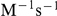	*M*	*K*	1.0-1.2e+04^c^
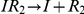	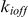	2.00e-01		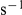	*K*		2.0e-01^c^
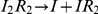	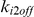	4.00e-01		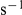	*K*		4.0e-01^c^
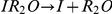	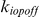	1.00e+00		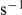	*K*		0.5-1.0e+00^c^
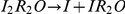	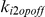	2.00e+00		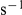	*K*		1.0-2.0e+00^c^
**Inducer transport**							
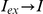	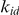	2.33e-03		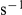	*K*		2.3e-03-1.4e-01^d^
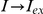	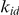	2.33e-03		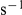	*K*		2.3e-03-1.4e-01^d^
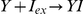	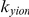	3.03e+04		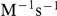	*K*		–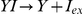	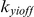	1.20e-01		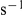	*K*		–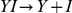	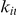	1.20e+01		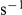	*K*		1.2e+01^e^

a *S* = *in vivo* single molecule experiment, *K* = *in vitro* (kinetic) experiment, *M* = model parameter fit to single-molecule distributions.

b [92],

c [74], [75],

d [69], [93],

e [70].

#### Lac operon regulation: Activation and inactivation of transcription

The regulatory behavior of the *lac* circuit results from the binding of the repressor to the *lac* operator, thereby inhibiting transcription initiation. There are three possible inducer–repressor species and we modeled the binding and unbinding reactions of each to the operator:
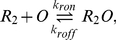
(1)

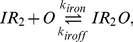
(2)

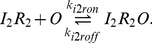
(3)


The stoichiometry of inducer–repressor binding is currently subject to debate [64]; it is unclear whether the affinity of 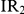 for the operator is of the same order as that of 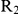 or much lower. We therefore compared the effect on our model of both a high 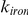 (comparable to 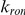) and a low 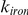 (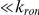). In either case, the affinity of 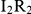 for the operator is thought to be low and we assumed 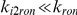 and 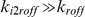. Values for the rate constants were obtained by fitting the model with experimental LacY distributions from single cells, as presented in Results.

#### Transcription, translation, and degradation

In the cell both transcription and translation are multistep processes involving numerous intermediates. Knowledge of the rate constants for each step of these processes *in vivo* is limited. We therefore assumed that each process was controlled by a single rate-limiting event and used pseudo first order rate equations with effective transcription and translation rates in the model. Such an approximation is reasonable as long as i) the concentration of the transcription and translation machinery is high and constant and ii) the non-Poissonian time delays missed by modeling such multi-step processes as a single step are not significant. For our model these conditions appear to be satisfied as the components of transcription and translation are among the most abundant in the cell and even though some, such as ribosomes, diffuse quite slowly there should nevertheless be a ready supply available at all times. Also, the times delays in these processes are on the order of seconds [27], while the dynamics of the cell response (here determined by the protein lifetime) is on the order of an hour.

Transcription of a LacY messenger RNA (mRNA) (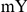) from the *lac* operon was modeled as a first order process dependent on a free operator:
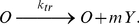
(4)


The effective transcription rate constant (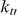) was a free parameter determined during model fitting.

Decay of and translation from 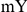 were modeled as a competition between RNase E enzymes [65] and ribosomes for an 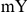
's ribosomal binding site (RBS). The rate of degradation of 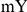 by RNase E was chosen to result in a mean lifetime (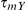) of 90 s, as reported by Yu *et al.* [21]. The effective translation rate was chosen to produce a mean of four LacY proteins over the lifetime of an average 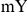 messenger, also as reported in [21]:
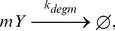
(5)

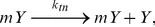
(6)with the effective rates given by 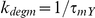 and 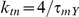.

The loss of membrane proteins in *E. coli* is primarily from dilution as a result of cellular growth over the cell cycle [66]. Therefore, degradation of LacY was modeled as a first order reaction with a half-life corresponding to the cell doubling time (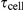),
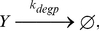
(7)where 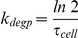.

#### Lac repressor/inducer kinetics

LacI rapidly dimerizes with very high affinity and the dimers further associate to form tetramers with a 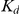 in the nanomolar range [67]. The tetrameric form enhances repression by binding multiple *lac* operators simultaneously [68]. However, modeling the DNA loops formed by this process would require additional inactive kinetic states, and as the focus of the current study is a two-state switch, we assumed a mutant form of LacI that did not tetramerize. This assumption allowed us to connect our model to single cell data from *lac* operator mutants incapable of DNA looping [22]. Furthermore, we assumed that the dimerization 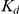 was sufficiently low that LacI only existed in the dimer state, the species 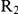. Ten molecules of 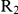 were placed in the cell, and we assumed the cell regulated this number to be constant, so that the noise from the transcription/translation of the repressor gene was ignored. Although noise from expression of LacI has been shown to have an effect on the induction rate [63], we chose to ignore this effect here to focus on the noise originating in the circuit itself.

The inducer molecules, isopropyl 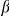-D-1-thiogalactopyranoside (IPTG) and thiomethyl-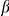-D-galactoside (TMG), are small sugar-like solutes that can either passively diffuse or be actively transported by LacY across the cellular membrane using an electrochemical proton gradient for energy. Inducer molecules in the extracellular space (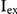) and those in the intracellular space (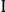,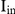) can diffuse across the membrane freely in both directions. The diffusive influx and efflux are modeled as first order reactions with the same kinetic constants,
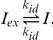
where 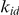 = 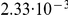 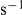 [58], [69]. The active transport of inducer molecules into the cell from the extracellular space is modeled as an irreversible Michaelis-Menten reaction,
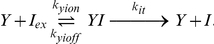



The value of 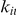 has been reported to be 12 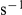 [70] and the Michaelis constant 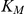 for the reaction with TMG to be ∼500 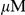 [71]. Similarly, the intracellular TMG concentration has been reported to be ∼70-fold higher than the extracellular concentration in fully induced cells [63], [72]. Under pseudo steady state conditions, the ratio of 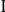 to 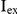 is related to 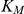 by the expression 
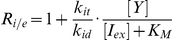
. In our model a maximal enrichment ratio of 70 corresponded to a 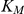 of 400 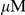, which is the value we used for TMG. The active influx of inducer molecules through the membrane is given by the standard Michaelis-Menten expression 
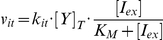
, with 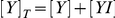 and 
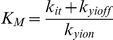
. However, for simulations of stochastic kinetics, we need unique values for 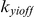 and 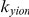. As long as the relationship between 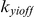 and 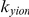 corresponds to the required 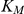 value, the influx of inducer will be correct irrespective of the values used. Only fluctuations in the external environment will be affected by the choice. In particular, a large value for 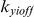 relative to 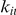 would require a correspondingly large value for 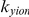, which would produce many unproductive inducer binding events outside the cell. These small fluctuations in the external inducer environment are expected to have little to no effect on gene expression dynamics inside the cell, but can have a significant impact on simulation cost. In the absence of any experimental data regarding the actual kinetic rates, we chose to make the 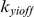 rate be 1% of the 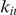 rate to improve simulation performance by minimizing the calculation of such non-productive binding events. The value of 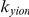 was then fixed by the relationship 
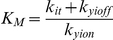
.

Upon entering the cell, inducer molecules can bind to free LacI and the repressor-operator complex, albeit with a much lower affinity. As each LacI monomer binds a single inducer molecule, there are three possible repressor dimer species, 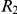, 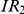 and 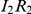, which interconvert according to the following reactions:
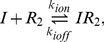
(8a)

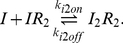
(8b)


*In vitro* kinetic data suggests non-cooperative binding (Hill coefficient of 1) of inducer to 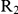 in the absence of *lac* operator DNA [64], [73], [74], corresponding to 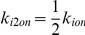 and 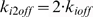 (see Supporting Text S1).

Although there is some equilibrium data suggesting the binding to the complex is cooperative with a Hill coefficient of 1.45 [74], such cooperativity was not observed in kinetic measurements of binding and unbinding [75]. For simplicity, a non-cooperative model was assumed:
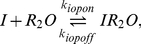
(9a)

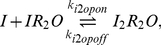
(9b)with 
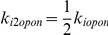
 and 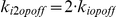.

**Figure 3:**
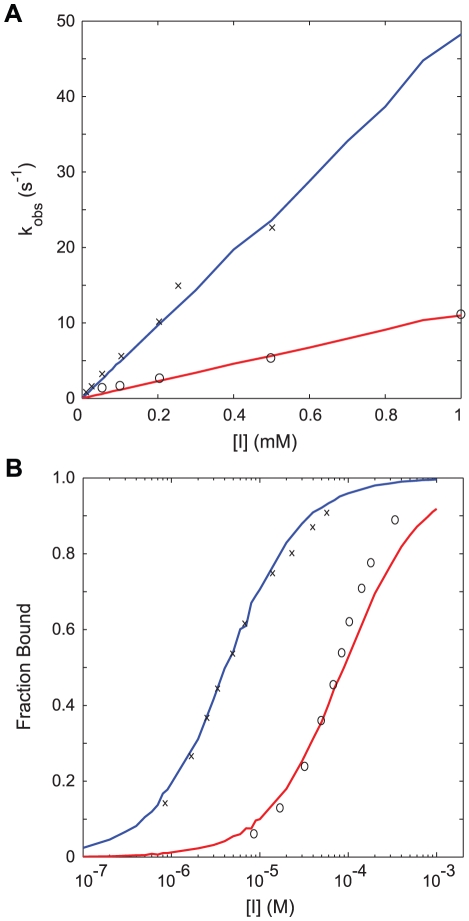
Fits of rate constants for IPTG binding to the *lac* repressor. (A) Pseudo first order rate constants observed during stochastic simulations of IPTG binding to (blue) repressor and (red) repressor-operator complex. At each inducer concentration 1000 simulations starting with 2 free (or operator-complexed) repressor dimers in a volume of 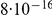 L were performed. The mean fraction of free repressor monomers as a function of time was fit to a single exponential to obtain the observed rate constant for binding at the inducer concentration. x and o are data from Dunaway *et al.* [75]. (B) Equilibrium binding of IPTG to (blue) repressor and (red) repressor-operator complexes. In a stochastic simulation at each inducer concentration, 20 free (or operator-complexed) repressor dimers in 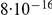 L were first equilibrated with inducer to reach the steady state. Following, 5 minutes of data were collected from which the equilibrium fraction of inducer bound repressor monomers was calculated. x and o are data from O'Gorman *et al.* [74].

Experimentally, different aspects of the *lac* circuit have been investigated using the inducers IPTG and TMG. To make use of the data one has to take into account the differences in their 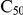 value, defined as the inducer concentration at which half of the LacI monomers are bound with an inducer. Kinetic and equilibrium binding measurements [74], [75] were available for IPTG binding to both free repressor and the repressor-operator complex. From the kinetic measurements, the rate constants for inducer binding and unbinding were 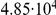 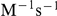 and 0.2 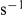 for the free repressor and 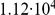 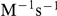 and 1.0 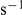 for the repressor-operator complex. This yielded a 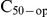 for binding to the repressor-operator complex (89 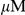) that is ∼20 times higher than for free repressor (4.1 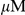). Figure 3 shows the results of using these rate constants in stochastic simulations of inducer binding; good agreement between simulations and experiments were seen for both kinetic and equilibrium measurements. TMG has been reported to have a 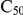 for binding to free repressor greater than that for IPTG by a factor of ∼10 [76]. However, since neither kinetic data nor detailed equilibrium studies were available, we assumed the same unbinding rate constants for TMG as IPTG and left binding to both the free repressor and repressor-operator complex as free parameters in the model to be fitted from single molecule experimental data.

### Well-stirred and spatially resolved simulations

Since the stochastic switch model is more complex than can be solved using analytic methods, we used computational Monte Carlo methods to sample the master equation and estimate the probability distributions. Two stochastic approaches were used to simulate the *lac* kinetic model: a well-stirred method using the CME and a spatially resolved method based on the reaction-diffusion master equation (RDME). The RDME model of the *lac* circuit can be thought of as a superset of the CME model in that all of the kinetic rates used for modeling reactions in the CME based model are also used in the RDME model, but with additional parameters regarding the spatial localization of particles and their diffusion in three-dimensional space.

#### Well-stirred model

The well-stirred model was sampled using a version of Gillespie's SSA algorithm [16] implemented in CUDA and running on the GPU. The *lac* model includes independent volumes for the extra- and intracellular space. These were tracked separately during the simulations and, to balance the flux of inducer across the membrane at equilibrium, the internal and external volumes were taken to be equal. Inducer in the extracellular space was maintained at a constant concentration for the propensity calculations by holding the number of 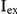 molecules fixed. Unless otherwise noted, we ran 10,000 replicates of each simulation to obtain sufficient sampling of the probability distributions.

#### Spatial model

The RDME model was sampled using our lattice microbe method. The multi-particle, *in vivo* diffusion operator for this method, based on that of Karapiperis and Blankleider [77], has been presented previously [37] and the reaction operator is provided in Supporting Text S1. Complete implementation details will be presented in a forthcoming publication. The method discretizes space onto a three-dimensional lattice of uniform spacing and time into uniform time steps. Chemical species randomly diffuse and react on the lattice according to rules defined using spatially dependent stochastic rate constants. A virtual microbe is constructed on a lattice by placing particles and obstacles on the lattice and specifying the reaction and diffusion properties of the lattice sites to mimic the spatial organization of the cell. The lattice microbe method uses the GPU as a computational coprocessor and the whole-cell simulations performed for this study were run for one hour of simulation time (slightly longer than a cell cycle of 55 min) using a time step of 50 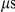 and a lattice spacing of 16 nm. For each external condition, 100 independent cell-scale simulations were run. Simulations were carried out on the NCSA Lincoln Intel 64 Tesla Cluster containing two NVIDIA Tesla GPU accelerators per node. Approximately 200 GPU-hours were required per hour of simulation time. Simulations of a smaller volume using 5 ns time steps and 2 nm lattice spacing were also performed to examine the variation in repressor rebinding as a function of packing density at the operator site.

Spatial models were developed for two *E. coli* phenotypes: fast- and slow-growth. For the fast-growth cells, the cellular volume was constrained to a typical *E. coli* cell shape: a cylinder with spherical end caps 2 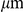 long by 0.8 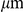 in diameter. The volume was surrounded by an impermeable cytoplasmic membrane separating the extracellular environment from the cytoplasm. We excluded the outer membrane from the model as its permeability is not thought to be a limiting factor for inducer transport. The intracellular space was randomly filled with stationary, *in vivo* obstacles to 50% volume fraction approximating the *E. coli* intracellular environment (see Table 2). The model was then coarse-grained onto a 16 nm resolution lattice, as described in Supporting Text S1.

**Table 2:** Obstacle abundance in *in vivo* spatial models.

			Fast Growth*^a^* ^,*b*^		Slow Growth*^b^*	
Type	Radius (nm)	Mass (kDa)	Count	% Vol	Count	% Vol
Ribosome	10.4	2700	35005	17.8	3021	5.7
Generic Protein	5.2	346	290908	18.6	96992	25.5
"	4.3	186	18610	0.7	6205	1.2
"	4.1	162	9907	0.3	3303	0.4
"	4.0	156	59862	1.7	19959	2.4
"	3.8	133	50261	1.2	16758	1.8
"	3.5	107	47365	0.9	15792	1.3
"	3.4	91	140212	2.5	46748	3.6
"	3.0	67	162894	2.0	54311	2.8
"	2.7	46	226358	2.0	75470	2.7
"	2.3	29	321118	1.8	107064	2.3
"	1.7	11	163939	0.4	54659	0.6
DNA*^c^*	–	89	0	0	31000	8.8

a Based on data from Ridgway *et al.* [38].

b Total occupied volume (excl. DNA) of 50%.

c Per cylindrical persistence length 2 nm in diameter and 50 nm long.

For the slow-growth phenotype, we based the spatial model on cryoelectron tomography (CET) of an *E. coli* cell undergoing slow growth. The full cell was approximately 3 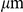 long by 0.4 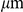 wide and tomograms encompassed approximately one-third of the cell length. Ribosomes were matched and located in the tomograms as described previously [50] as was a portion of the cytoplasmic membrane. The position of the missing membrane was extrapolated to form a contiguous surface and ribosomes were placed at their measured positions in lieu of random placement. The completed one-third cell model was then mirrored to produce the opposite cell pole. The middle third was randomly generated using ribosome densities from the adjacent CET data. Ribosomes were not observed in the central volume of the cell, which we inferred to be the condensed nucleoid. A random walk algorithm was used to place a full-length *E. coli* chromosome in the nucleoid region. Starting from the center of the nucleoid region, cylinders representing DNA persistence lengths 50 nm in length and 2 nm in diameter were randomly added end-to-end such that the angle between successive cylinders was constrained to 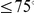. If the random walk left the nucleoid region, the path was unwound a number of steps and a new random path started. This process was repeated until all 31,000 persistence lengths had been added. Since later coarse graining of the model onto a 16 nm resolution lattice spread out the nucleoid density, we did not constrain the chromosome to be circular in this model. Following nucleoid addition, the non-ribosomal *in vivo* obstacles were proportionally placed in the model at random locations (including within free space in the nucleoid region) to reach an occupied volume fraction of 50% (Table 2). The final slow growth model was then coarse-grained to a 16 nm lattice for simulation.

In *E. coli*, translation of an mRNA containing the sequence for an integral membrane protein is thought to be coupled with translocation of the resultant protein across the cytoplasmic membrane by the Sec translocase [78], *i.e.*, cotranslational translocation. Specifically, LacY has been observed to require the bacterial signal recognition particle (SRP) pathway for functional membrane integration [79]–[81]. However, it is not presently clear whether or not transcription and translation of membrane proteins are also coupled such that the gene being transcribed is also physically located near the site of translocation. For this reason, we modeled two variants of operator placement for comparison: in the fast-growth phenotype the operator site was located in the center of the cell and in the slow-growth phenotype the operator site was placed on the nucleoid near the membrane (∼32 nm away) close to a cell pole. These two different configuration allowed us to compare the mRNA dispersions for close versus far gene–translocation distances.

Messenger 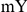 molecules were created at the location of the operator following transcription and then allowed to diffuse in the cytoplasm with a diffusion constant of 0.1 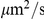 [82], [83]; in the slow growth model 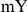 molecules were precluded from entering the nucleoid. In the spatial model, then, 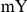 was required to diffuse to the membrane before translation could occur; Equation 6 was limited to membrane sites. Since ribosomes likely attach to an mRNA's RBS while transcription is still ongoing [84], the model assumed that 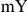 molecules were protected from degradation by RNase E until after 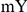 reached the membrane; Equation 5 was also limited to membrane sites. Translation of 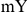 produced LacY proteins at the same location as the mRNA in the membrane. LacY molecules were constrained to diffuse in the membrane with a diffusion coefficient of 0.1 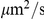.


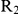
 molecules were randomly placed in the cell and diffused at 1 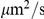 within the intracellular volume. Small inducer molecules diffuse at ∼1000 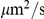 in extracellular space and ∼100 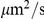 in intracellular space. However, in order to reach simulation times on the order of the cell cycle the maximum diffusion coefficient in the model, which depends on the lattice spacing and time step, was 1.28 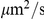. Therefore, the diffusion coefficient of the inducer molecules was set to 1.28 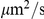. Since inducer molecules are present in large numbers and they diffuse faster than the repressor this approximation was not expected to have a noticeable effect. The lattice was connected to a infinite reservoir of 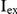 molecules through the use of constant concentration boundary conditions to maintain the extracellular space at a constant inducer concentration.

### Maximum likelihood fitting of gene expression models

We analyzed the capability of the burst and two-state analytic models of gene expression to recover parameters from our stochastic simulations of an inducible switch by fitting molecular distributions. We used a maximum likelihood method to estimate the model parameters. Briefly, the likelihood 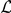 of the model parameters 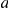 having produced a set of observations 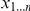 is given by
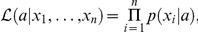
where 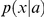 is the conditional probability of observation 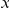 occurring given the parameters 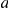. The parameters that maximize this likelihood function are those that describe the best fit of the model to the data, assuming a uniform prior distribution for the parameter probabilities. To find the best parameters for a model of gene expression, 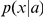 was calculated using the model's steady-state probability density function with the 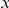 values being the protein counts from the 10,000 simulations. The parameter values that minimized the negative log of the likelihood function were then found using downhill simplex minimization as implement in the Matlab fminsearch function. We estimated the confidence intervals for different sample sizes by taking 1000 random sets of either 50 or 200 cells from the full set of 10,000 and performed maximum likelihood estimation on each of these data sets. The confidence range for each parameter was then defined by the middle 95% of the values obtained during these random resamplings.

The burst model was first expressed in terms of parameters 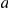 and 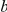 by Friedman *et al.* [24] as the Gamma distribution. However, since our stochastic simulations produced discrete protein counts, we used the discrete formulation for the steady-state probability density derived by Shahrezaei and Swain [25] in terms of a negative binomial distribution 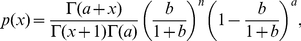
(10)with parameter 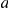 being the burst frequency (bursts per mean protein lifetime) and 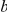 being the burst size (proteins produced per burst).

The two-state model was fit using the steady-state probability density function derived by Shahrezaei and Swain [25]:
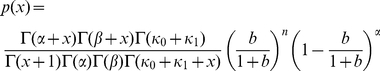
(11)

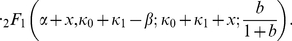
(12)


In this expression the parameters are 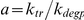, 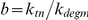, 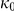 (the activation rate), and 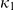 (the inactivation rate), the latter two being expressed in units of mean protein lifetime. Additionally, 
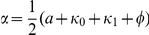
, 
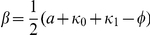
, 
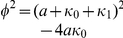
, and 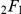 is Gauss's hypergeometric function. Fitting with all four parameters free often resulted in convergence in a local minima, so we adopted a fitting procedure whereby we first constrained the 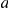 and 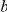 parameters and fit only 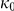 and 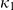 to obtain initial estimates of these two parameters. In the fully induced state 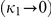 the above probability density function reduces to a negative binomial distribution with no dependence on 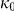 or 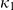, only 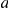 and 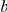. Since neither 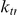 nor 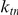 depend on inducer concentration, it is a reasonable approximation to use the values for 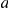 and 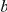 in the fully induced state as initial estimates for all inducer concentrations. After obtaining an initial fit for 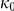 and 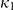, we then performed another fit with 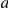 and 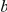 unconstrained and with 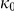 and 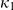 allowed to vary ±5%. This procedure resulted in convergence at a higher likelihood score than when all four parameter were fit simultaneously for all distributions except one.

## Results

Here we present the result of our study into the noise effects in the inducible *lac* genetic switch. The first two sections describe the fitting of model free parameters to data from single-molecule fluorescence studies on *E. coli* populations. The next two sections analyze noise in the well-stirred circuit due to its regulatory control elements. The final two sections report on changes to the behavior of the circuit from *in vivo* effects, using a model of a spatially heterogeneous, crowded cell and then an experimentally determined cell structure under an alternate growth phenotype.

### Linear relationship between transcriptional burst size and inducer concentration

In a recent *in vivo* single-molecule fluorescence study, Choi *et al.* measured the distributions of a fluorescent reporter protein under control of the *lac* operator in individual *E. coli* cells at various inducer (TMG) concentrations [22]. They performed the measurements in the absence of LacY's positive feedback by replacing its gene with that of the membrane protein Tsr in the *lac* operon. This enabled an accurate determination of the protein distribution produced by the circuit at a given inducer concentration without any confounding non-linear effects due to enhancement of the internal inducer concentration by LacY. In the absence of DNA looping, they were able to fit their observed distributions to a gamma distribution 
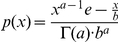
, where 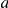 was interpreted as the frequency of transcriptional bursts relative to the protein lifetime and 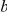 as the mean number of proteins produced per burst. They observed a relatively constant value for the burst frequency of 3–4 and a linearly increasing relationship between burst size and inducer concentration at low to intermediates concentrations.

**Figure 4:**
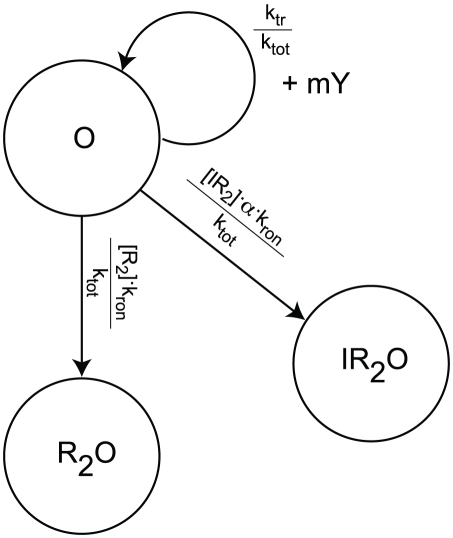
Markov diagram for transcriptional bursting in the *lac* circuit. Under low-to-moderate inducer concentrations, a burst begins when the operator enters the 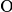 state and ends when it transitions to a repressor bound state. 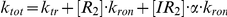.

To understand the origin of the linear relationship between burst size and inducer concentration and to reproduce this behavior in our model, we derived an expression for the burst size as a function of kinetic parameters in our model. As long as bursts are infrequent relative to protein degradation, *i.e.* once a free operator is bound with a repressor it remains bound for a significant fraction of the cell cycle, transcriptional bursting from the *lac* operon can be modeled as a Markov process with competition between RNA polymerase (RNAP) and the various LacI species for binding to the free operator (see Figure 4).

Transcription initiation by RNAP was modeled as a pseudo first order process (Equation 4), with a rate constant of 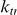. The two repressor states with potentially significant binding affinity were 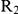 and 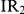, shown in Equations 1 & 2. Free repressor binds with free operator with a rate constant of 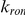 resulting in a pseudo first order rate of 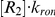. Given the current debate surrounding the binding affinity of the 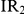 state to the operator, we set the rate constant 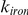 to be proportional to the free repressor binding constant and analyzed the effect of varying the proportionality constant 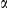 on the pseudo first order rate 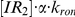. This model of transcriptional bursting assumes that the binding of 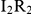 to the free operator is negligible at low inducer concentrations by assuming 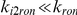 and ignoring Equation 3. In practice, this condition was satisfied when 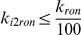. We used the upper limit 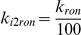 in our model, which is within the range experimentally reported [76].

Following the unbinding of a repressor from the repressor–operator complex, the probability of transcription initiation (and subsequent mRNA creation) occurring at the free operator as opposed to a repressor re-binding is
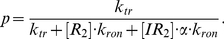
(13)


The probability of a given number of consecutive transcription initiation events (the size of the mRNA burst) then follows a geometric distribution with 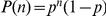 of which the mean is 
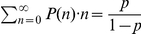
. However, repressor unbinding events that produce no mRNA are not observable as a burst, therefore the mean number of mRNA produced in a transcription bursts (B) is
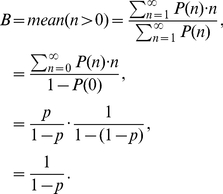
(14)


Combining Equations 13 and 14 gives the expression for the mean transcription burst size in terms of the rate constants for transcription initiation and repressor binding
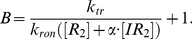
(15)


Given the inducer mass balances (see Supporting Text S1) and the expression for the total number of repressor dimers 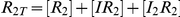, one can derive the equilibrium concentrations of the two repressor species
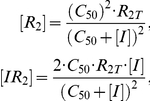
where 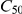 is the inducer concentration at which half of the repressor monomers are bound to an inducer molecule. Substituting 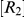 and 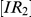 into Equation 17 gives the expression for the transcription burst size as a function of inducer concentration
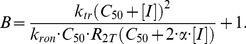
(16)


**Figure 5:**
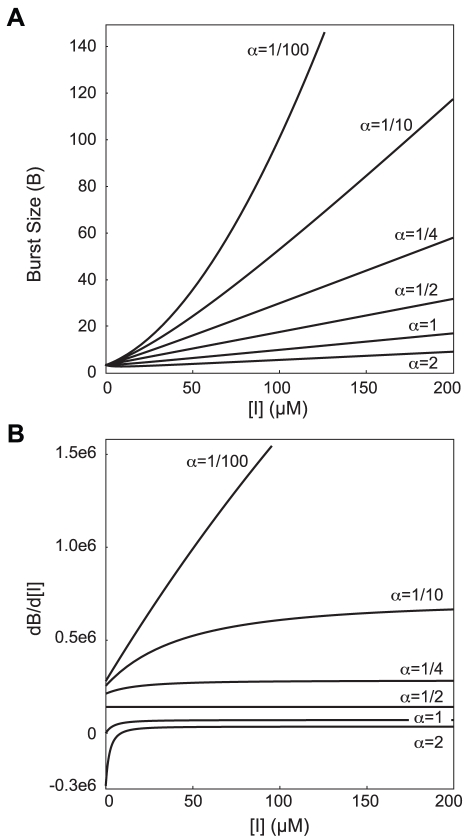
Parameter space of repressor binding parameter 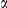. (A) Mean burst size as a function of inducer concentration for various values of 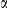, where 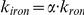. Parameters used were 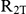 = 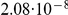 M, 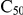 = 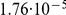 M, 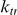 = 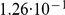 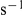, and 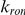 = 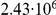 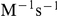. (B) The rate of change in the burst size with respective to the inducer concentration.

From this last equation it is clear that the transcription burst size will be linear over the entire range of inducer concentrations only when 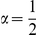. Figure 5 shows the effect of varying 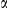, of particular interest are the very low values. When 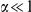, the transcription burst size does not linearly increase over the range of inducer concentrations for which this behavior has been reported (0–200 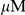). In the model here formulated, a linear relationship between size and inducer concentration exists only when the binding affinity of 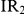 for the free operator is comparable to that of 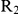. For our simulations, we therefore chose 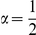, such that 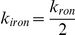, as this value assumed no effect on the unbound repressor monomer due to a single bound inducer and gave a strictly linear relationship for all inducer concentrations.

### Fitting transcription and inducer/repressor rate constants to single-cell distributions

**Figure 6:**
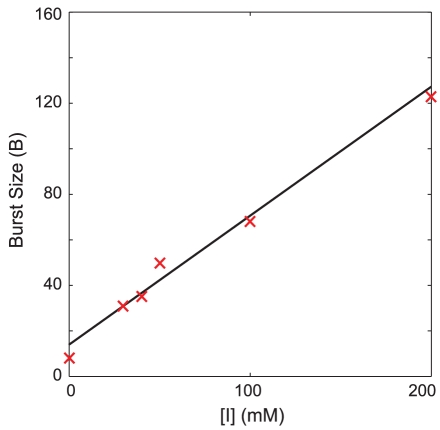
Linear fit of burst size to inducer concentration. x are data from Choi *et al.* [22].

To obtain values for the model parameters 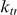, 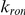, and 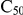, we used the distributions for LacY reported by Choi *et al.* [22], specifically the inferred burst frequency (bursts per cell cycle) and size parameters (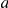 and 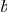) from their gamma distribution fits. From Equation 18, the mean transcription burst size as a function of inducer concentration is 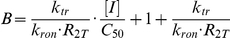. This equation is linear in inducer concentration and by fitting it (multiplied by the mean number of proteins produced per mRNA) to the experimental protein burst sizes, as shown in Figure 6, one can constrain the kinetic parameters. The y-intercept of the line fixes the ratio of transcription to repression in the uninduced state (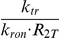) and the slope can then be used to obtain 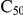 = 17.6 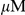 for TMG.

The linear fit, however, only fixes the ratio between 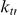 and 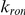. To recover unique values for these two rate constants, we next considered the mean duration of each transcription burst. The interpretation of the shape parameter 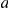 of the gamma distribution as the burst frequency is only meaningful if the burst duration is short compared to the protein lifetime. In that case, individual exponentially sized bursts can be considered exponentially distributed in time and therefore act independently to give rise to a gamma distribution of protein abundance. In setting rate constants for the model, then, we wanted to ensure that the burst duration was appropriately short.

**Figure 7:**
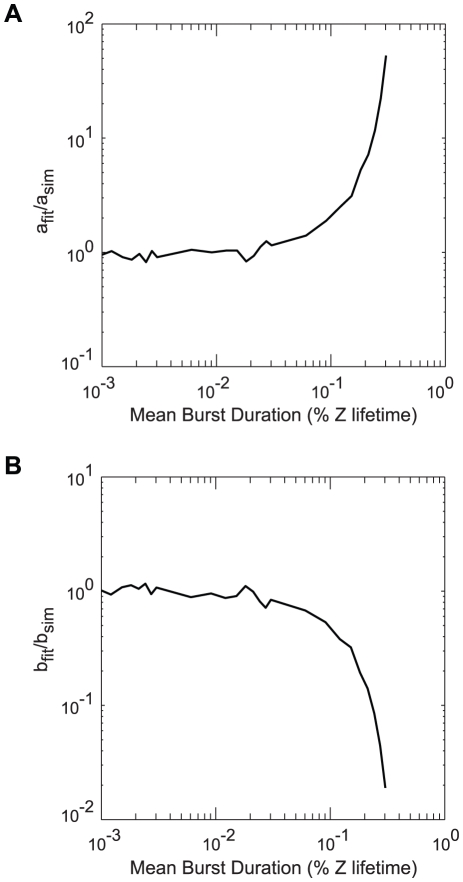
Burst analysis of stochastic simulations of a simple two-state process. The two-state process was described by: 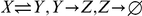. Rate constants were chosen such that on average 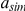 bursts of Z with a constant burst size 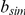 were produced during Z's mean lifetime with the mean duration of each burst lasting for the indicated fraction of the lifetime. At each point, 250 stochastic simulations were run until the probability density was stationary and then the distributions of Z were fit to gamma distributions to obtain the 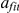 and 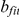 parameters. The ratios of (A) 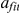/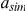 and (B) 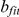/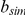 as a function of the burst duration show the range of burst durations for which a gamma distribution fit can reliably recover the original parameters. In this example 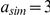 and 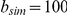.

The burst duration 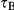 is simply the mean time for a repressor to bind to a free operator. Given a constant 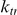, a linear relationship between burst size and inducer concentration also implies a linear relationship between 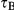 and inducer concentration as can be seen from
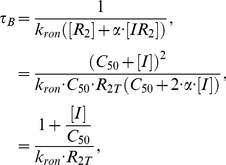
(17)where 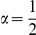 in the last step. For TMG, the linear relationship between burst size and inducer concentration extended to at least ∼200 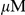, which is ∼11 times the 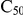 value for TMG of 17.6 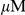. From Figure 7 it can be seen that the interpretation of 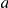 as the burst frequency begins to break down once 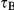 is >5% of the protein lifetime. Using 5% of the protein lifetime as 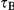 for 200 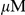, we can compute the value for 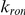 that gives the appropriate 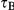: 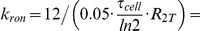 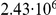 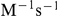, using a cell doubling time 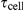 of 55 minutes. With this value for the repressor binding rate, a single repressor molecule in an *E. coli* cell would take ∼200 s to find a free operator. This is somewhat faster than the 354 s reported by Elf *et al.* [51]. Using the above value for 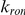 and the ratio of 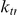 to 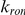 from the linear fit of the experimental data we obtained the value for the transcription rate 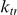 = 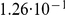 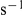. This rate for transcription initiation resulted in a steady state concentration of ∼2500 LacY molecules per cell in the fully induced state, within a factor of two of the ∼1000–1200 reported in the literature [22], [26]. The value also falls within the range of 1000–3000 seen for other highly expressed proteins in *E. coli* [85]. Accurate measurements of the burst duration in the *lac* system, particularly in the fully induced state, would increase the accuracy of our model.

**Figure 8:**
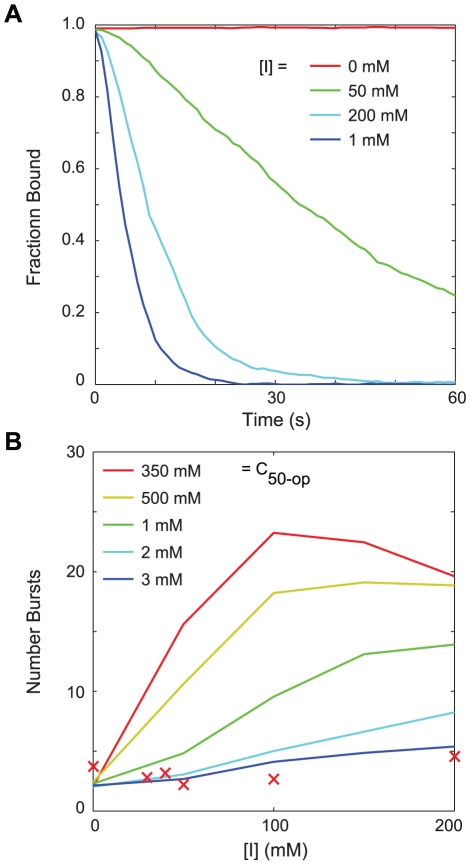
Parameter fitting for inducer–repressor–operator interactions. (A) Fraction of operator regions bound by a repressor as a function of time following an increase of IPTG to the indicated concentration. In these simulations, 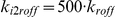. (B) Number of bursts over the mean protein lifetime as a function of inducer concentration for a variety of values of the 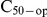 parameter. x are data from Choi *et al.* [22].

In order to reproduce a burst frequency of 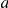 over the mean LacY lifetime in the model, the repressor should dissociate from the operator with a frequency 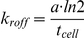, assuming that each dissociation event produces a burst and that 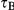 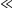 the cell cycle. The burst frequencies inferred by Choi *et al.* for TMG levels ≤100 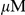 are relatively constant with a mean of ∼3 bursts. This corresponds to 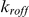 = 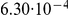 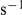. Since the dissociation of a repressor dimer is not thought to be significantly affected by the binding of a single inducer molecule, 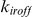 = 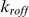. The affinity of a repressor dimer with two bound inducer molecules, however, is thought to be much lower, *i.e.*, the binding of a second inducer molecule essentially knocks the repressor off of the operator. In the absence of this effect, the response to an increase in inducer concentrations would take a significant fraction of the cell cycle. Elf *et al.* reported a response time of <60 seconds for addition of IPTG to concentrations from 50 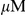 – 1 mM [51]. Therefore, we fit 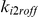 such that the response of the model to increase in IPTG agreed with the published data. The best fit value was obtained for 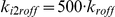 (shown in Figure 8A).

The final kinetic rates to be defined were those regarding the binding of TMG to the repressor–operator complex (Equations 10 & 11). As discussed in Methods, we used the same dissociation rates as for IPTG, leaving only the association rates 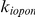 and 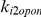, both of which can be derived from the 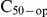 value, which is the inducer concentration at which half of the repressor–operator complexes have a bound inducer. Figure 8B shows the effect of varying 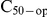 on the burst frequency. As 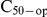 approaches 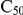, the burst frequency begins to diverge from its expected value. This is due to the increasing occupancy of the 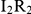
O state, which can decay much more quickly into a free operator than the other repressed states; with operator free more often, there are more bursts over the lifetime of a protein. A value of 3 mM for 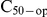 gave the best agreement with the experimental burst frequencies for TMG.

### Population distributions without positive feedback

**Figure 9:**
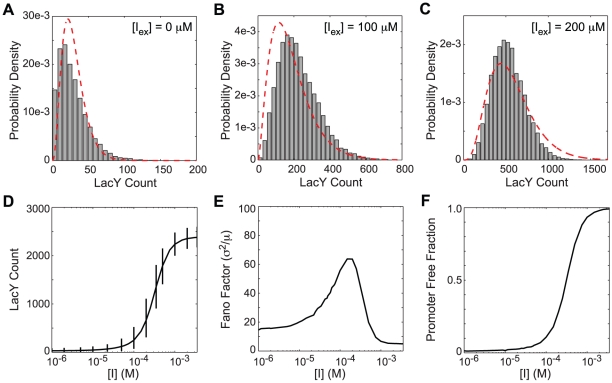
Steady state LacY distributions from the well-stirred NPF model. Distributions at inducer concentrations of (A) 0, (B) 100, and (C) 200 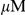 TMG. Shown are (gray bars) histograms from 10,000 Gillespie trajectories and (red dash) gamma distributions from Choi *et al.* [22]. (D) Mean LacY as a function of inducer concentration along with 95% ranges. (E) The noise in the LacY distributions as quantified by the Fano factor (variance over the mean). (F) The fraction of time spent in the transcriptionally active state.

Using the derived rates, we performed well-stirred stochastic simulations of the *lac* model in the absence of LacY positive feedback (NPF model), obtaining the stationary LacY distributions as a function of internal inducer concentration shown in Figure 9. Compared to the intrinsic noise of the two-state model, the NPF model contains additional noise contributions from the non-constant rates for transitioning between active and inactive transcriptional states. The distributions showed the widest cell-to-cell variability due to the intrinsic noise of the system at intermediate inducer concentrations of 50–400 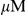. At high inducer concentrations the population migrated toward a less variable distribution, as expected. Up to 100 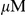, the population distributions agreed well with those reported by Choi *et al.* but at 200 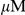 the agreement began to break down. This discrepancy at concentrations >100 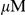 was caused by two primary factors: the burst duration and the action of inducer knocking repressor off of the operator. Increasing the repressor binding rate would improve the fit by decreasing the duration of each burst, but would cause a large increase in the total number of LacY molecules in the fully induced state, which is not supported experimentally. Alternatively, one could increase the 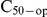 value, causing less inducer instigated dissociation of the repressor–operator complex, but this would decrease the responsiveness of the circuit to addition of inducer, which is also not supported experimentally. Clearly, in order for the model to have greater predictive power, additional features would be necessary. For example, adding a delay between production of mRNA to account for the steps of RNAP open complex formation or more detailed modeling of translation. But lacking the *in vivo* experimental results to validate any additional complexity, we chose to ignore these effects and analyzed the model as described.

The gene regulation function (GRF) of an genetic system describes the relation between the activity of a gene and its regulatory control elements [86]–[88]. In the steady state, protein production is balanced by protein degradation/dilution. The mean protein count as a function of the control elements provides a method to analyze a GRF. The mean number of LacY per cell as function of the TMG concentration (Figure 9D) and the fraction of time spent in the transcriptionally active state (Figure 9F) show the regulatory behavior of the NPF model. We saw a typical sigmoidal regulatory response that was well fit by a Hill equation with an inflection at 312 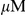 and a Hill coefficient of 2.11. In a stochastic system, though, the mean rate of gene expression is just one piece of information. As important for a stochastic GRF is how the distribution changes with inducer concentration. The Fano factor (variance/mean) provides a measure of the variation of the distribution. For reference, the Fano factor of a Poisson process is 1. For the NPF model (Figure 9E) the Fano factor monotonically increases until 100–200 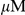 where it peaks at a value of ∼60 and then begins to decrease ending at a lower value of relative noise than at zero inducer.

### Noise due to positive feedback

Next we investigated noise in the inducible genetic switch when the positive feedback regulatory link was active (PFB model). The *lacY* gene located in the *lac* operon codes for the integral membrane protein LacY, which actively imports inducer molecules (lactose/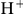 co-transport) establishing a positive feedback loop as shown in Figure 1C. The presence of active LacY in the membrane creates a concentration gradient enriching the intracellular environment with inducer molecules relative to extracellular space. For a fixed 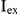 concentration, the underlying GRF for the *lac* operon therefore operates not only at an increased inducer concentration but, since the number of LacY is different for each cell, across a distribution of internal inducer concentrations.

**Figure 10:**
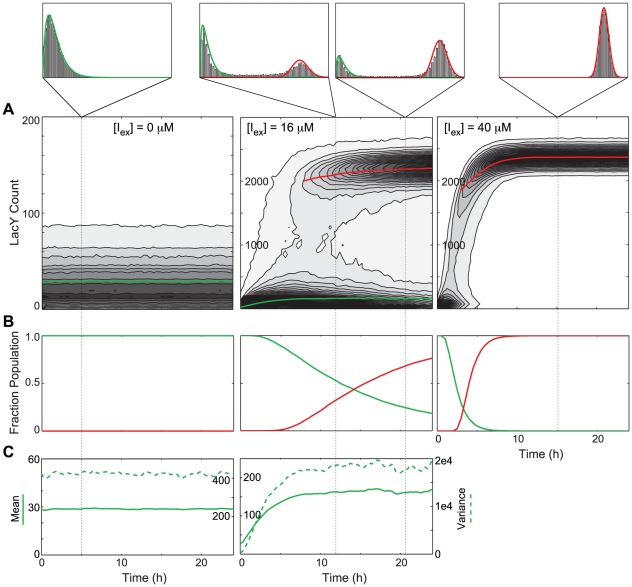
Response of an uninduced PFB population to the addition of external inducer. (A) Probability density 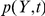 (arbitrary units, darker = higher) of the number of LacY in a cell over the course of 24 hours. Shown are representative responses for populations in the uninduced range (0–10 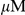; left), the bimodal range (10–25 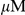; center), and the concerted induction range (>25 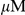; right). Lines show the mean value of the (green) uninduced and (red) induced subpopulations. (B) Fraction of the cells in each of the subpopulations. (C) The (solid) mean and (dotted) variance of LacY in the uninduced subpopulation.

We calculated the population distributions for the PFB model using well-stirred stochastic simulations at various 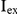 concentrations. Starting from a stationary population distribution in the absence of inducer, each population of 10,000 cells was subject to an instantaneous increase in 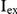 and simulated for twenty-four hours. Above an  concentration of ∼10 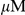, cells in the population began to switch to an induced state in which LacY expression was near its maximum value (see Figure 10A and B). Above ∼25 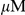 the transition to full expression was relatively concerted throughout the population. In the range of 10–25 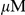, though, there were two transiently stable subpopulations, one uninduced and the other induced – the overall population was bimodal for a time.

To quantify the switching behavior of the population, we classified cells at regular time intervals as uninduced with <400–600 LacY (best fit for each 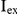) or induced with >1750 LacY. Each subpopulation was then analyzed separately. The mean and variance of the distributions (Figure 10C) show that, after an initial response phase, the distribution of the uninduced subpopulation was stable over time. This was true even as the total number of cells in the uninduced population was decreasing as cells within it were switching to the induced state. At intermediate inducer concentrations, the uninduced cell population appeared to reach a stationary distribution from which cells independently and stochastically transitioned to the induced state. In contrast, at higher inducer concentrations the population migrated as a whole in a more downhill-like manner.

**Figure 11:**
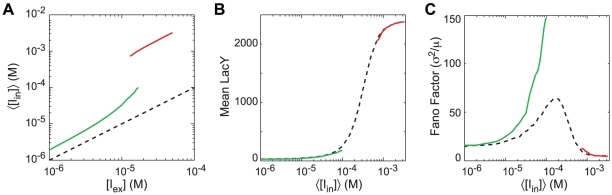
Effect of positive feedback on GRF noise. (A) Mapping of the mean internal inducer concentration for a given external concentration for the (green) uninduced and (red) induced subpopulations. (black dotted) The values for the lac circuit without positive feedback are shown for reference. (B) The mean number of LacY in the subpopulations as a function of internal inducer concentration. (C) The noise in the LacY distribution.

Noise in a GRF can be expressed in terms of its effect on the phenotypic variance in a population under identical environmental conditions. To compare noise between the NPF and PFB models, we first mapped 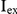 concentrations to mean 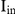 concentrations in the uninduced and induced subpopulations (in the NPF model 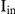 = 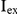). We then compared both the mean of the LacY distributions and the Fano factor for the two models. The mean values for the LacY distributions (Figure 11) were similar but the noise in the uninduced subpopulation was significantly higher in the model with positive feedback. Since the underlying GRF is equivalent between the two models, it is the action of the GRF on the distribution of 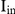 concentrations that gives rise to the increase in intrinsic noise in the PFB model.

### Differences in circuit behavior due to *in vivo* crowding

Having established the well-stirred PFB stationary distribution, we next evaluated the effect of *in vivo* molecular crowding on the distributions, the PFB+IV model. One obvious reaction subject to spatial effects is the rebinding of the repressor to the operator following an unbinding event. Immediately after unbinding, a repressor is necessarily localized near the operator, *i.e.* it has a memory of its location. As was shown by van Zon *et al.* [27], this memory effect increases the probability of repressor rebinding at very short times compared to a well-stirred approximation. Previous studies only considered the effect of normal diffusion following unbinding but there is an additional effect caused by anomalous diffusion due to *in vivo* crowding.

**Figure 12:**
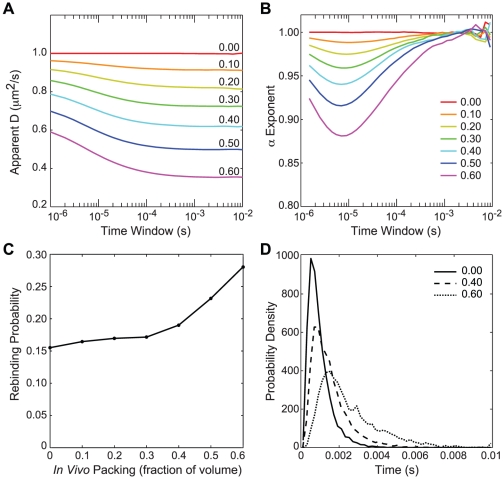
The effect of *in vivo* crowding on repressor rebinding. Each line represents the mean of 5000 trajectories. (A) The observed diffusion coefficient, 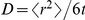, as a function of time scale for a repressor diffusing in a volume with the indicated fraction occupied by *in vivo* obstacles. (B) 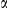
–exponent arising from fitting 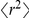 to a model of anomalous diffusion, 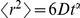. (C) The probability for a repressor to rebind with the operator before diffusing into the bulk (64 nm from operator) following unbinding, as a function of the *in vivo* packing. (D) The distribution of escape times for repressors that diffuse to bulk rather than rebind, at three packing values.

To investigate repressor rebinding in an *in vivo* environment, we performed reaction-diffusion simulations of a 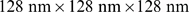 volume centered on an operator immediately following unbinding of a repressor. We varied the packing density of the approximated *in vivo* environment to study its effect on rebinding. Figure 12A and B shows that there is an anomalous effect at short time scales (<1 ms). Repressor diffusion at very short time scales is normal at the *in vitro* rate, but between 1–100 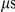 there is a period of anomalous behavior, and at very long time scales repressor diffusion returns to normal diffusion behavior with a lower diffusion coefficient D. Brownian dynamics simulations of proteins in a virtual *in vivo* environment [39] show a similar anomalous behavior when including only steric constraints with a minimum in the time exponent of ∼0.8 for proteins slightly larger than the 75 kDa repressor dimer. When electrostatic effects are included in the Brownian dynamics simulations, however, the apparent diffusion coefficient as well as the anomalous exponent change greatly, so our results should only be considered an upper bound on the *in vivo* effects. Including further electrostatically driven interactions such as non-specific binding, will increase the anomalous behavior of the repressor.

The anomalous behavior of the repressor causes it to spend more time near the operator following unbinding than would be expected for purely Brownian diffusion, leading to more encounters with the operator and a potentially greater probability of rebinding. To measure the change in rebinding probability, we counted the number of repressors that rebound to the operator following unbinding versus the number that escaped into bulk solution, defined here as leaving the simulation volume. As can be seen in Figure 12C, as the density of *in vivo* crowding increases, the probability of rebinding goes up. Compared to an *in vitro* unpacked environment at 15% probability of rebinding, at 50% packing the probability of rebinding is ∼24%. The distribution of escape times also broadens (Figure 12D) with particles in general taking longer to diffuse away. The anomalous memory effect resulted in the duration of some bursts being significantly shorter than expected.

**Figure 13:**
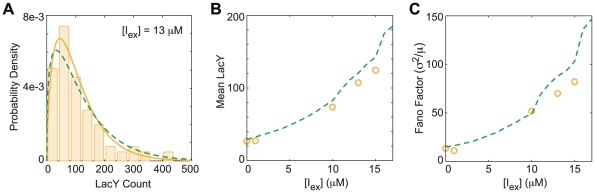
LacY PFB+IV *in vivo* distributions. (A) The distribution of LacY in (orange bars) 100 modeled *E. coli* cells at 13 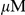 TMG concentration compared with (green dotted) the PFB well-stirred distribution. (B) Mean number of LacY proteins in the (circles) PFB+IV and (green dotted) PFB models. (C) The noise in the distributions.

To study the effect of burst duration differences on the stationary LacY distributions in a population, we used our lattice microbe method to generate PFB+IV trajectories of spatially resolved rapid-growth *E. coli* cells (see Methods). Beginning with the stationary distribution from the well-stirred PFB population, 100 cells were simulated at five internal inducer concentrations for one hour, slightly longer than the duration of a cell cycle (55 minutes), see Video S1. Over the course of the simulations, distributions in the *in vivo* models gradually migrated to lower mean values and lower noise, as can be seen in Figure 13. Two factors caused this migration: First, the shorter burst durations due to the anomalous diffusion effect described above resulted in fewer proteins being produced per burst and more time spent in the inactive state led to more frequent bursts and less noise. Second, the effective increase in repressor due to the decreased reaction volume. In contrast to spatial effects in an *in vitro* environment [27], it appears that *in vivo* crowding lowers both the mean value and the noise in distributions of observables. Since bacterial cells such as *E. coli* are known to have packing density changes during different portions of the cell cycle and/or growth conditions, this presents the possibility of measuring these *in vivo* effects on living cells if the observable distributions can be accurately quantified as a function of the cell cycle or growth conditions.

### Whole-cell modeling using experimentally determined cell architecture

**Figure 14:**
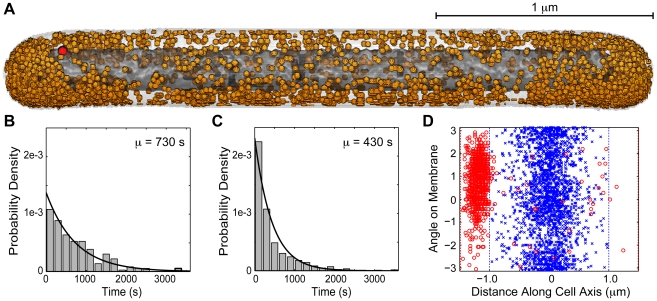
Analysis of cryoelectron tomography based cell model. (A) Slow growth *E. coli* cell model based in part on data from a tomographic reconstruction. Shown are (orange) ribosomes, (light gray) membrane, (dark grey) condensed nucleoid, and (red) *lac* operator. (B+C) Distribution of repressor–operator complex lifetimes for the fast and slow growth models, respectively. Curves show fits to an exponential distribution with the given mean. (D) Position of mRNA–membrane contact after diffusion of mRNA produced at the *lac* operon in (blue x) fast growth and (red o) slow growth models. Dotted lines show the length of the respective cells.

As a first attempt at addressing how changes in the cellular environment due to growth conditions affect gene expression noise, we used CET of *E. coli* cells under slow growth to build a whole-cell model of an individual bacteria (Figure 14A). Under conditions of slow growth in minimal media *E. coli* B/r K grows as elongated cylinders with diameter ∼400 nm [89], which are amenable for CET [50]. The tomograms were used to identify the membrane-enclosed volume of an individual cell along with the three-dimensional position of ribosomes within it. The *E. coli* B/r K cell under slow growth had only 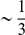 of the volume of typical fast growing cells. A central region of the cell was devoid of ribosomes and inferred to be the location of the condensed nucleoid.

We studied the operation of the *lac* circuit in the slow-growth phenotype (PFB+IV+CET) using 100 random replica cells. Each replica used the same experimentally measured cellular geometry and ribosome positions, but a random distribution of other molecules including a condensed chromosome (see Methods for details). Cells were simulated using the lattice microbe method in 15 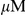 external TMG, starting with LacY and mRNA counts sampled from the uninduced stationary distribution of the well-stirred PFB model, see Video S2. Simulations were run for either one hour or until the cell had induced, whichever came first. There were clear differences between the slow- and fast-growth *in vivo* models. Of the 100 slow-growth cells, 11 induced within one hour whereas only a single fast-growth cell induced in the same time period. Also, the mean number of LacY molecules in the uninduced slow-growth population increased ∼15% over the course of one hour, compared to the fast-growth population which decreased ∼15%.

Analysis of the simulation trajectories revealed that the primary cause of the differences in LacY distributions between the slow- and fast-growth models was an increased mean inducer concentration in the smaller cells, 100 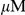 versus 42 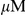. For a given number of LacY proteins, the cells with the smaller volume had an increased internal inducer concentration. The increased levels of inducer caused a slight lengthening of the mean duration of free operator events, 68 seconds versus 64 seconds, and a corresponding larger burst size. A bigger change was observed in the mean lifetime of the repressor–operator complex, which decreased to 430 seconds from 730 seconds (Figure 14B,C). The decrease effected an increase in the mean number of transcription bursts per hour, to 4.3 from 2.6.

The slow-growth model provides a first approximation as to the effect of differences in cellular architecture on stochastic gene expression. The model assumed the same number of repressor molecules for smaller cells, which may not be accurate as repressor is known to regulate its own expression. However, since the largest effect was due to an increased rate of repressor unbinding due to elevated inducer levels, which is independent of repressor concentration, we consider the general result of increased burst frequency and rate of induction in smaller cells to be intriguing. It implies that there might be a difference in the switching properties during the first part of the cell cycle following division when a large burst of LacY would have an increased influence on switching due to the reduced cellular volume. Such an effect could potentially be measured using cell synchronization techniques. Although specific ribosome placement likely also influenced repressor rebinding in the slow-growth model, any differences were overshadowed by the effect of the cell volume change. Nevertheless, in a situation where the placed macromolecules are involved in the reaction kinetics, we anticipate accurate (non-uniform) placement will take on much greater importance.

Another large difference between the slow- and fast-growth models arose due to the presence of a condensed nucleoid coupled with the smaller cell diameter. In the fast-growth cells the chromosome was assumed to be diffuse and not an obstacle for mRNA diffusion. In the slow-growth cells, the chromosome was randomly placed in the ribosome-excluded region observed in the tomograms and it represented an obstruction for mRNA diffusion. Additionally, the operator was positioned in the center of the fast-growth cells and at the edge of the nucleoid in the slow-growth cells. As can be seen in Figure 14D there was a dramatic increase in localization of mRNA in the slow-growth cells as a result of this arrangement. A recent report of mRNA localization in bacteria [90] suggests that the relative locations of transcription and translation in bacteria may indeed be correlated. If that is generally true, then in systems where the location of protein synthesis affects the reaction kinetics it will be important to know the actual position of the gene in the cell and measurement of the dispersion of the transcripts might be one way to quantify whether the gene is physically located near the site of translation and translocation.

## Discussion

### Fitting population distributions to models of stochastic gene expression

Fitting protein population distributions to gene expression models will be a key step in developing simulations of other stochastic cellular systems with predictive power. Parameters obtained from fitting the distributions will drive the computations. Our stochastic simulations of the inducible *lac* switch provide an opportunity to test the process of extracting parameters from a population distribution arising from a complex gene expression system using simplified but analytically tractable models. To do so, we fit the stationary population distributions from our simulations to both the burst and two-state models (Figure 1A & B) and evaluated their capability to recover the stochastic rate constants used in the simulations (*e.g.* 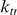, 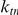, etc). The analysis was performed for each of the different noise variations described above, corresponding to the NPF, PFB, and PFB+IV simulations. We excluded the PFB+IV+CET simulations from this study as they were not performed over a range of inducer conditions. The best fit parameter values were obtained by maximum likelihood estimation using the stationary probability density function (PDF) for the burst and two-state models, Equations 12 & 14 in Methods. Fits were performed using 10,000 cells for NPF and PFB simulations and 100 cells for PFB+IV simulations.

**Figure 15:**
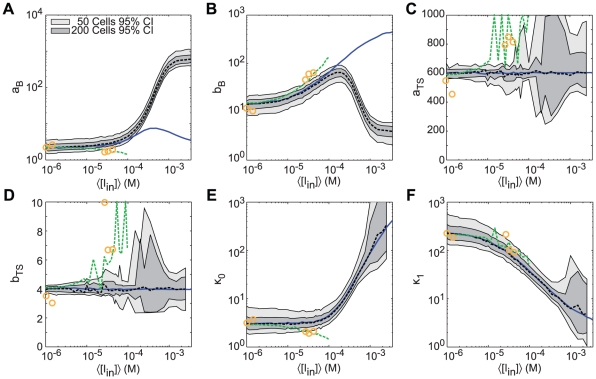
Maximum-likelihood fitting of two models for gene expression to stochastic simulations of an inducible genetic circuit. (A and B) Parameter fits from the burst model. (C–F) Parameter fits from the two-state model. Shown are fits for (black dotted) NPF simulations, (green dotted) PFB simulations, and (orange circles) PFB+IV simulations. Also shown are (blue solid) actual parameter values calculated from the simulation data. Shaded areas indicate the 95% confidence intervals for ML fits using distributions from 50 and 200 NPF cells.

Figure 15A & B show parameter estimates obtained from fitting using the burst model's gamma distribution PDF (Equation 12). The 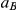 and 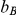 parameters (the B subscript indicates parameters for the burst model) reliably recover the burst frequency and burst size, respectively, in the NPF simulations at low inducer concentrations, but diverge from the simulation values above ∼100 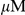. This is as expected as the model is only valid when the duration of each burst is short enough that sequential bursts can be considered as occurring independently, <5% of the protein lifetime as shown in Results. In particular the divergence occurs near the switching threshold, making this model most suitable for analyzing the system in the uninduced state with low expression levels. However, the clearness of the biological interpretation for the model parameters as the burst frequency and size make the model extremely valuable over the regime it is valid.

Fitting the NPF simulation data to the stationary PDF of the two-state model (Figure 1B; Equation 14) provides good parameter estimates over a wider range of inducer concentrations. The fits are shown in Figure 15C–F for the parameters 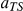 (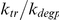; the TS subscript indicates two-state), 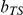 (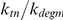), 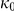 (the rate constant for operator activation), and 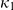 (the rate constant for operator inactivation), respectively. As the inducer concentration increases, though, many more cells are required to obtain reliable estimates. Using even 10,000 cells, we were unable to obtain good fits for the highest expression levels. At these inducer levels so little time is spent in the inactive state that the difference in likelihood values for different switching rates is insufficient to find a unique maximum using 10,000 samples. However, as the time spent in the inactive state approaches zero (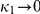) the probability distribution approaches a negative binomial distribution without dependence on 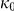 or 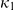, so it is possible to estimate the 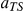 and 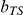 parameters in the fully induced state by fitting to a negative binomial.

The two-state model therefore appears to be a reasonable method for fitting the NPF simulations. Using the fitting parameters (along with known or estimated mRNA and protein degradation rates), one can readily recover the transcription and translation rates as well as the rates of the operator switching between active and inactive states at a given inducer concentration. Even though switching between active and inactive states in the *lac* switch is not a first order process – it is controlled by 14 reactions – at a given inducer concentration the steady state switching times are reasonably well-approximated by a single exponential. A further improvement in the two-state model would allow 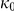 and 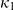 to depend on the inducer concentration using, *e.g.*, a Hill function. An analytic solution to such a model would allow extraction of parameters from a multivariate fit using data across all inducer concentrations. However, to the best of our knowledge, the analytic form of such a model has not been derived.

Using the steady state distributions from the PFB simulations, neither model achieves good fits. For the two-state model, the 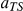 and 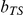 parameters are recovered correctly, but the fits for the 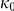 and 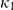 parameters are lower than expected. The poor fit for these parameters is due to noise in the switching rates of the cell population caused by differences in internal inducer concentrations. With positive feedback, it will be very difficult to reliably estimate model parameters from population distributions due to non-linear noise. Fitting to experimental data should be done in the absence of positive feedback, such as by using gene knock-outs to eliminate circuit components responsible for positive feedback. However, if an analytic model were developed including positive feedback effects, comparison of systems with and without these effects could provide estimates of positive feedback parameters, *e.g.* inducer transport rates.

Fits to the PFB+IV simulations as well show deviations from the expected values; *in vivo* crowding noise changes the parameter fits. For these simulations, an additional source of discrepancies with the models is the non-Poissonian behavior of repressor rebinding – there is a positional memory in the system for a short time following unbinding. In our simulations the effect from *in vivo* conditions due to excluded volume is modest, but there are other *in vivo* factors still not accounted for in them, especially non-specific binding as recently reported by McGuffee and Elcock [39], which would have an even larger effect on repressor rebinding. Also, repressor rebinding most likely occurs via a series of 1D sliding and 3D hopping steps, the effect of which on rebinding in a crowded environment is not known. Accounting for *in vivo* effects when deriving parameter from experimental population distributions, which would include *in vivo* noise contributions, will be difficult. Possibly an iterative process of refinement may be required, starting with model estimates and proceeding through multiple rounds of spatial simulation.

Overall, it appears that fitting population distributions to the two-state model could prove to be an effective way of obtaining rate constants for stochastic simulations of gene regulation. Single-molecule *in vivo* fluorescence imaging provides a way to experimentally measure these distributions. Measurements over a range of regulatory conditions could then be used to build a stochastic gene regulation function, provided the actual probability distributions from single-molecule experiments were available at each condition. However, it is important to acknowledge that our simulations did not include a contribution from global extrinsic noise. Noise in our simulations under conditions of high expression approaches Poissonian, as expected from the intrinsic noise of an uncorrelated random process. A recent study has clearly shown, though, that there is a constant level of global extrinsic noise in gene expression in *E. coli*, maintaining population heterogeneity even at high levels of gene expression [85]. This implies that a way to correct for the global extrinsic noise will be needed in order to fit experimental population distributions at high expression levels.

### Probability landscape of an inducible *lac* switch

**Figure 16:**
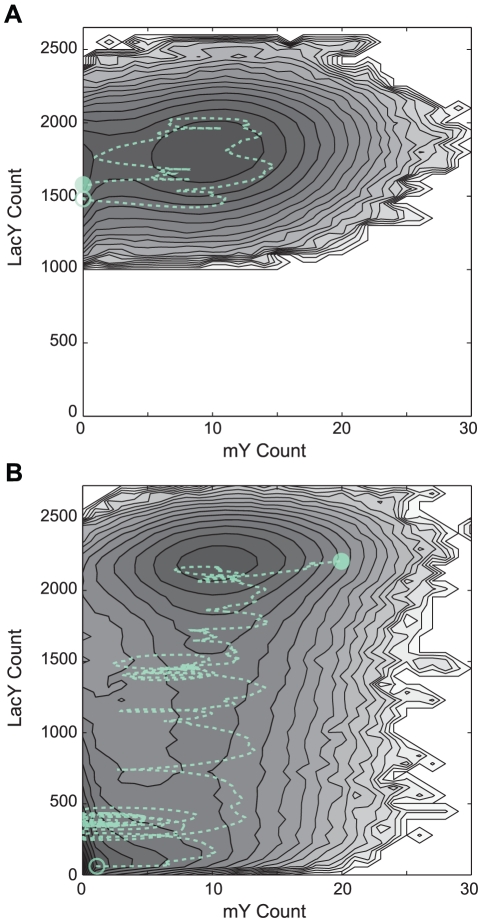
Probability landscape of protein–mRNA abundances in the inducible *lac* switch model. (A) Steady-state probability landscape (arbitrary units, darker = higher) for the NPF model at 500 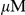 TMG. The dotted line shows the trajectory of a representative cell during a ∼3 hour interval starting at the open circle and ending at the closed circle. (B) Probability landscape of the PFB circuit over a period of 24 hours following the addition of external TMG to 16 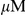. The line follows a single cell switching from the uninduced to the induced state over the course of ∼13 hours.

The probability distribution for a stochastic biochemical system to be in a particular state represents the totality of information about the system. From it various measures of the behavior of the system such as the mean first passage time between two states or their relative population at the steady state can be obtained. For models of stochastic gene expression, two relevant reaction coordinates are the number of protein and mRNA molecules in the system. We used our stochastic simulations to reconstruct the two-dimensional probability landscapes (negative log of the PDF) of the NPF and PFB models at two external TMG concentrations (Figure 16).

The steady-state landscape of the NPF simulations at 500 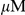 inducer shows a bistable mRNA distribution that has been reported by others [25], [91]. One minima is located near 0 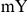/1700 LacY and the other near 10 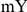/1800 LacY. Note that the stable 10/1800 point does not imply that 180 LacY were produced per 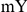, as the degradation rates of the two molecules differ. At 500 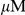 inducer, the net time some cells stochastically spend in the inactive state is greater than the typical lifetime of the mRNA bursts. These cells then drift to a zero mRNA abundance. The higher density is caused by an accumulation of these cells near the zero mRNA level until their next mRNA burst pushes them back into a random cycle around the mean mRNA burst size. Interestingly, though, the protein distributions at the two mRNA minimum are different, with the protein abundance being slightly lower in the lower mRNA minimum. This means that the protein and mRNA probability distributions are not completely independent of each other; the joint probability distribution has cross terms. While not a large difference, it is nevertheless possible that the joint protein–mRNA distribution could be used to obtain better parameter fits for the two-state model with fewer cells, if the mRNA counts were known.

The bimodal distributions seen in the LacY distributions from the PFB simulations (Figure 10) are recapitulated in the probability landscape for switching. The two-dimensional landscape allows classification of both the uninduced and induced states in terms of their relative protein and mRNA abundances. Additionally, the landscape reveals the transition path for switching from the uninduced to the induced state. One can imagine two possible scenarios for the transition, either the gradual build-up of protein by a series of small bursts, or alternatively, by the random occurrence of a small number of larger bursts. For the *lac* system with DNA looping, Choi *et al.* [26] have persuasively argued for the random large bursts as the switching initiator. The switching mechanism of the stochastic system in the absence of looping, however, is not so clear. The probability landscape of our *lac* model suggests that it is actually the occurrence of several large mRNA bursts, on the order of >10 molecules, in quick succession that is responsible for putting the cell on the path to induction. Cells can spend a significant amount of time in a high LacY but low 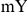 state without inducing. This behavior is apparent in the cell trajectory plotted in Figure 16B. Switching therefore is a process in which not only a protein threshold must be crossed, but also an mRNA threshold.

### Conclusions

Our goal with this study was to go beyond previous stochastic simulations of the *lac* circuit by using information from single molecule protein distributions and experimentally determined cellular architecture to constrain the kinetic parameters and estimate the effect of spatial heterogeneity on the response of the switch. The kinetic model of the inducible *lac* genetic switch presented in this study illustrates the utility of incorporating single-molecule, single-cell data when modeling cellular biochemical systems. The model was derived using a kinetic framework reproducing a linear relationship between protein burst size and inducer concentration at low concentrations, as has been reported experimentally. Analysis of the linear relationship in terms of inducer–repressor–operator interactions suggests that the stoichiometry of repressor binding is such that repressor dimers with one bound inducer still have significant affinity for the *lac* operator. Furthermore, single-cell population distributions were used to obtain estimates of the effective rate constants for transcription and repression in the cell. With future increases in performance of the lattice microbe simulation method it should be possible to iteratively refine the kinetic rate constants to account for the effects of cellular architecture, such as we obtained here from CET experiments, and cytoplasmic crowding. Using such *in vivo* adjusted rate constants the *in vivo* models should then more accurately reproduce experimental population distributions, which are after all measured under *in vivo* conditions, than the well-stirred models.

The *lac* model without positive feedback provided a baseline for the noise in the regulation of the *lac* operon. Intrinsic noise at low gene expression was significantly higher than Poissonian and peaked when the promoter was active 10–30% of the time. The model with positive feedback produced similar mean values for a given intracellular inducer concentration, but the noise was substantially greater. We attribute this effect to the non-linear gene regulatory function operating on a distribution of intracellular inducer levels. Global extrinsic noise in the transcription/translation machinery is a large contributor to population heterogeneity at high levels of expression, but we excluded such noise from the current study.

Fitting of data from stochastic simulations of the *lac* switch with the burst and two-state models of gene expression showed both the potential and limitations of these models to interpret stochastic gene regulation. The burst model described the data well under conditions of low expression, when the gene was active for ≤5% of the mean protein lifetime, but diverged for increasing expression levels. The two-state model better described the data at higher levels of expression, but near full induction the error in the activation and inactivation rates became significant. Additionally, the fits provided estimates of the number of cell measurements necessary to produce reliable parameter estimates. With 50 cells the worst-case relative error was ±90%, but with 200 cells it dropped to ±32%. Fitting to joint mRNA–protein distributions might improve parameter estimation. Fits to data with positive feedback indicated that both models were unable to reliably extract parameters from populations with such feedback.

Switching of cells from the uninduced to the induced state was observed in the positive feedback model without DNA looping over a range of low inducer concentrations. During switching, the uninduced population maintained a stable stationary distribution while cells stochastically transitioned to the induced population. The probability landscape showed that both an mRNA and a protein threshold must be crossed for a cell to switch to the induced state. The probability landscape for the DNA looping case is likely different, but additional model states would be required to accurately represent DNA looping.

Finally, we have presented what we believe to be the first whole-cell simulations of stochastic gene expression using experimentally obtained cellular architecture. These simulations showed that *in vivo* conditions can impact the stochastic noise in biological systems. Positional memory of transcription factors following unbinding, amplified by anomalous diffusion due to molecular crowding, introduces non-Poissonian statistics. In the case of our *lac* model in fast-growth cells, this effect caused a decrease in the mean value of the LacY distribution by up to 10% and its noise by up to 20%, for a given environmental condition. In a slow-growth cell phenotype we saw a large increase in burst frequency due to the smaller cell size, as determined from cryoelectron tomography. From this difference we infer that changes in cellular size and/or shape during the cell cycle can have an impact on stochastic processes. Since spatial noise can vary from cell-to-cell or even during the cell cycle so we consider it a type of extrinsic noise. The necessary computational resources and experimental data are becoming available such that computational biologists should consider adding spatial degrees of freedom into physical models of cellular biochemical networks.

## Supporting Information

Text S1 Supporting methods. Further methods describing the derivation of the rate constant relationship for non-cooperative ligand binding, the lattice microbe reaction operator, and the lattice coarse-graining technique.
(PDF)

Video S1Simulated colony of *E. coli* cells responding to inducer. Video composite of trajectories from six spatial PFB+IV simulations at 15 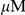 inducer. Yellow circles are LacY proteins and red circle are mY mRNA molecules. Two cells begin the process of switching to the induced state.(MOV)

Video S2Trajectory of a PFB+IV+CET cell responding to inducer. Visualization of a single slow-growth CET modeled cell responding to 15 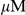 inducer. Gray spheres are ribosomes and the blue region the nucleoid. Yellow circles are LacY proteins and red circles are mY mRNA molecules. The repressor–operator complex is green and the free operator is white.(MOV)
