# Supplementary material for: Noise Contributions in an Inducible Genetic Switch: A Whole-Cell Simulation Study
Source: PLoS Comput Biol. 2011 Mar 10;7(3):e1002010. doi: 10.1371/journal.pcbi.1002010 (PMC3053318; doi:10.1371/journal.pcbi.1002010)
Supplement: Text S1 — Supporting methods. Further methods describing the derivation of the rate constant relationship for non-cooperative ligand binding, the lattice microbe reaction operator, and the lattice coarse-graining technique. (PDF) [file pcbi.1002010.s001.pdf]

Noise Contributions in an Inducible Genetic Switch: A Whole-Cell Simulation Study

Roberts, Magis, Ortiz, Baumeister, Luthey-Schulten

Supporting Text S1

Supporting Methods

Derivation of rate constant relationship for non-cooperative ligand binding

Sequential binding of two ligands L to an enzyme with two binding sites E_2 can be described by the following two equations:

$$L + E_2 \underset{k_{off}}{\overset{k_{on}}{\rightleftharpoons}} LE_2, \tag{1}$$

$$L + LE_2 \underset{k_{2off}}{\overset{k_{2on}}{\rightleftharpoons}} L_2E_2. \tag{2}$$

From these one can obtain the following mass balance equations:

$$\begin{aligned}\frac{d[E_2]}{dt} &= -k_{on}[L][E_2] + k_{off}[LE_2],\\ \frac{d[LE_2]}{dt} &= k_{on}[L][E_2] - k_{off}[LE_2] - k_{2on}[L][LE_2] + k_{2off}[L_2E_2],\\ \frac{d[L_2E_2]}{dt} &= k_{2on}[L][LE_2] - k_{2off}[L_2E_2].\end{aligned}$$

Solving for the steady state, by setting these equations equal zero, and using the expression for the total number of enzyme molecules $E_{2T} = [E_2] + [LE_2] + [L_2E_2]$, it can be shown that the fraction of enzyme binding sites occupied by a ligand molecule is given by

$$f_B = \frac{(2[L] + K_{d2})[L]}{2([L]^2 + K_{d2}[L] + K_d \cdot K_{d2})}, \tag{3}$$

where the equilibrium dissociation constants $K_d = \frac{k_{off}}{k_{on}}$ and $K_{d2} = \frac{k_{2off}}{k_{2on}}$ have been used. The concentration of ligand resulting in half of the binding sites being bound is then

$$C_{50} = \sqrt{K_d} \cdot \sqrt{K_{d2}}.$$

Cooperativity can be described using the Hill equation, $f_B = \frac{[L]^n}{(C_{50})^n + [L]^n}$, where the Hill coefficient n is indicative of the degree of cooperation between multiple ligands L binding to a macromolecule. Formulating

ligand binding as a Hill equation with coefficient of 1 gives

$$f_B = \frac{[L]}{\sqrt{K_d} \cdot \sqrt{K_{d2}} + [L]}. \tag{4}$$

Equating Equations 3 & 4 it becomes apparent that Equations 1 & 2 are non-cooperative only when $K_{d2} = 4 \cdot K_d$. If the probability of ligand unbinding is independent of the number of inducers bound, the rate of unbinding when two ligands are bound will be twice that when a single ligand is bound: $k_{2off} = 2 \cdot k_{off}$. Therefore, $k_{2on} = \frac{k_{2off}}{K_{d2}} = \frac{2 \cdot k_{off}}{4 \cdot K_d} = \frac{1}{2} k_{on}$.

Lattice microbe reaction operator

Models of spatially inhomogeneous stochastic chemical systems are often described using the reaction-diffusion master equation (RDME). In the formalism of the RDME the system's volume is divided into a set of discrete subvolumes (commonly a lattice in numeric implementations) with the chemical species in the system being distributed amongst the subvolumes. Reactions occur only between species within a subvolume and each subvolume is considered to be well stirred such that the reactions follow standard kinetic theory (constant probability per unit time). Each subvolume is then well-described by the chemical master equation (CME). Diffusion is accounted for by random transitions of species between subvolumes also with constant probability per unit time. The time evolution of the probability P for the system to be in a specific configuration x (counts of every chemical species for each subvolume) starting from a given initial state x_0 at t_0 obeys the RDME:

$$\begin{aligned}\frac{\partial P(x,t)}{\partial t} &= \sum_{v \in V} \sum_{r=1}^{R} -a_r(x_v) P(x,t) + a_r(x_v - S_r) P(x - S_r 1_v, t) \\ &+ \sum_{i \in V} \sum_{j \in V} \sum_{\alpha=1}^{N} -d_{ij}^{\alpha} x_i^{\alpha} P(x,t) + d_{ji}^{\alpha} (x_j^{\alpha} + 1)^{\alpha} P(x + 1_j^{\alpha} - 1_i^{\alpha}, t)\end{aligned}$$

Here, x_v^{α} is the number of molecules of species α ($\alpha = 1, \cdots, N$) in subvolume v ($v \in V$). R is the number of reactions. a_r is the reaction propensity for reaction r in given the state of a subvolume x_v. S is the stoichiometry matrix. d_{ij}^{α} is the diffusive propensity for one molecule of species α to diffuse from subvolume i to j.

The RDME is difficult to analytically study for even simple systems, and instead is most often sampled using a Monte Carlo approach. Many independent realizations of the system's path through the probability space are computationally calculated and then combined to reconstruct the probability density function (PDF) of the RDME. Several implementations of RDME samplers have been published [1–6]. Our approach

is focused on sampling the RDME for systems that approximate the physical aspects of an individual cell, therefore we call it the lattice microbe method. Diffusion and reaction rate constants can be spatially dependent to allow the modeling of cellular features such as the membrane, nucleoid, and other cellular structures. Simulating spatially resolved stochastic systems is computationally costly because of the large number of calculations associated with tracking the diffusion of many particles. We take a brute-force approach by adopting a parallelizable RDME method involving timesteps and nearest neighbor transitions and perform the diffusion calculations using the GPU [7]. Uniquely, our approach is of sufficient performance to permit the inclusion of *in vivo* crowding into the model, by constructing an approximation of the crowded cytoplasm using reflective sites.

Computing a single realization of the system's RDME starts with the discretization of space into a three-dimensional cubic lattice of spacing λ and time into time steps of length τ. Then, starting from an initial state at t_0, the state of the system at times t_τ, $t_{2\tau}$, $t_{3\tau}$, ... is sequentially calculated. Each time step is broken into reaction and diffusion operations. The reaction operator $\mathcal{R}$ calculates any changes in the chemical species present in each subvolume over the time step and the diffusion operator $\mathcal{D}$ calculates any redistribution of the species to and from neighboring subvolumes:

$$N(\alpha, \vec{r}, t + \tau) = \mathcal{D} \cdot \mathcal{R}N(\alpha, \vec{r}, t),$$

where $N(\alpha, \vec{r}, t)$ gives the number of molecules of species α present in lattice site (subvolume) $\vec{r}$ at time t. The implementation of the diffusion operator has previously been described [7], here we focus on the reaction operator.

Chemical species can react during a reaction operation according to defined stoichiometry and kinetic rates. We assume a well-stirred environment in each subvolume during a timestep and calculate the probability per unit time of a reaction occurring in a Gillespie-like manner [8]. For the current study we constrained the time steps and reaction volumes such that there was a low probability (≤5%) for any particular reaction to occur in a subvolume during a given time step. This allowed us to assume that a maximum of one reaction occurred per time step per subvolume. The reaction operator calculates a random realization of all the possible reactions given the current state of a lattice site and the stoichiometry matrix S, which contains the changes in the counts of each of the S chemical species for all of the M reactions:

$$\mathcal{R}N(\alpha, \vec{r}, t) = N(\alpha, \vec{r}, t) + \sum_{m}^{M} S_{\alpha,m}\, \theta(m, \vec{r}, t).$$

The function $\theta(m, \vec{r}, t)$ is a stochastic function returning 1 if reaction m occurs in lattice site $\vec{r}$ during the time

interval t–$t + \tau$, otherwise 0. It function such that the probability of a reaction occurring is consistent with

$$P(\theta(m, \vec{r}, t) = 1) = \int_0^{\tau} a_m(\vec{r}, t) e^{-\sum_m^M a_m(\vec{r},t)\, t'} \, dt'$$

when $a_m(\vec{r}, t)$ is the Gillespie propensity (probability per unit time) of reaction m occurring in the subvolume $\vec{r}$ at time t.

Lattice coarse graining technique

The lattice microbe method simulates reaction-diffusion processes on a three-dimensional lattice representing the cellular environment. A key first step in performing a lattice microbe simulation is defining the three-dimensional cellular model, including the membrane, cytoplasmic obstacles, nucleoid, and other cellular features. The initial model is constructed in continuous space and then mapped onto a lattice at a given resolution, typically 2-16 nm. The model building algorithm uses a computational geometry data structure known as a kd-tree [9] to store the three-dimensional model. The kd-tree data structure is a binary search tree that efficiently stores objects in three-dimensional space and allows quick placement of new objects into unoccupied volume. This becomes particularly important when randomly placing the cytoplasmic obstacles, which at 50% crowding by volume amount to $\sim$1.5 million objects. Features in the cellular environment are mapped to the diffusion and reaction properties of lattice sites, such that particles diffuse and/or react differently based on the cellular architecture. For example, cytoplasmic obstacles are mapped as a group of lattice sites into which diffusion of particles is prohibited.

When the dimensions of a lattice site are larger than the size of the object being mapped onto the lattice, there is no longer a one-to-one or one-to-many mapping of objects to lattice sites. In that case, it is necessary to create a coarse-grained representation of the cell that omits the detailed description of the objects while preserving their overall effect. Many of the cytoplasmic obstacles in the *in vivo Escherichia coli* model are much smaller than the 16 nm lattice size used in the long-time simulations presented in the main text. The key contribution of these obstacles to the simulations is the effect they have on diffusion of particles: their presence causes diffusion in the cytoplasm to be anomalously subdiffusive. As such, the cell models for the long-time simulations were coarse-grained to 16 nm resolution in such a way as to preserve the anomalous behavior of proteins diffusing in the cytoplasm.

Anomalous diffusion can be phenomenologically described by the equation $\langle r^2 \rangle = 6Dt^{\alpha}$, where $\langle r^2 \rangle$ is the mean-square displacement, D is the diffusion coefficient, t is the time, and α is the anomalous exponent. For normal diffusion $\alpha = 1$ and for subdiffusion $\alpha < 1$. As shown in the main text, for a protein diffusing in a crowded environment α is one at short time scales, monotonically decreases to a given minimum value

dependent on the packing density, and then monotonically increases back to one at long time scales. A coarse-graining function that preserves anomalous diffusion, then, should minimize the error in the minimum value of the α exponent.

The coarse-graining function used in this study first determines the fraction of each site's three-dimensional volume that is occupied by obstacles in the continuous space model. Then, all of the lattice sites with occupancy greater than a specified cutoff are considered occupied and the remainder empty. To determine the proper cutoff value, simulations are performed using a range of cutoff values and the α diffusion exponent measured for each. The cutoff that minimizes the error in α relative to the expected value becomes the coarse-graining parameter for that particular lattice occupancy and spacing. For 16 nm lattice spacing and 50% packing using the *in vivo* obstacle distribution reported in the main text, the best cutoff was 0.19, *i.e.* all lattice sites with more than 19% volume occupied in the continuous space model were marked as obstructed. This corresponded to 37.6 % of the total number of sites in the coarse-grained model. This coarse graining method did result in a shift of the anomalous minimum to greater time scales, but the agreement with the α value at the minimum was good.

Supporting Video Legends

Video S1: Simulated colony of *E. coli* cells responding to inducer. Video composite of trajectories from six spatial PFB+IV simulations at 15 μM inducer. Yellow circles are LacY proteins and red circle are mY mRNA molecules. Two cells begin the process of switching to the induced state.

Video S2: Trajectory of a PFB+IV+CET cell responding to inducer. Visualization of a single slow-growth CET modeled cell responding to 15 μM inducer. Gray spheres are ribosomes and the blue region the nucleoid. Yellow circles are LacY proteins and red circles are mY mRNA molecules. The repressor–operator complex is green and the free operator is white.

References

[1] Hattne J, Fange D, Elf J (2005) Stochastic reaction-diffusion simulation with MesoRD. Bioinformatics 21: 2923–4.

[2] Rodríguez JV, Kaandorp JA, Dobrzyński M, Blom JG (2006) Spatial stochastic modelling of the phosphoenolpyruvate-dependent phosphotransferase (PTS) pathway in Escherichia coli. Bioinformatics 22: 1895–901.

[3] Marquez-Lago TT, Burrage K (2007) Binomial tau-leap spatial stochastic simulation algorithm for applications in chemical kinetics. J Chem Phys 127: 104101.

[4] Drawert B, Lawson MJ, Petzold L, Khammash M (2010) The diffusive finite state projection algorithm for efficient simulation of the stochastic reaction-diffusion master equation. J Chem Phys 132: 074101.

[5] Ferm L, Hellander A, Lötstedt P (2010) An adaptive algorithm for simulation of stochastic reaction-diffusion processes. J Comput Phys 229: 343–360.

[6] Arjunan SNV, Tomita M (2010) A new multicompartmental reaction-diffusion modeling method links transient membrane attachment of E coli MinE to E-ring formation. Syst Synth Biol 4: 35–53.

[7] Roberts E, Stone JE, Sepulveda L, Hwu WMW, Luthey-Schulten Z (2009) Long time-scale simulations of *in vivo* diffusion using GPU hardware. In: Proceedings of the 2009 IEEE International Symposium on Parallel & Distributed Processing.

[8] Gillespie DT (2007) Stochastic simulation of chemical kinetics. Annu Rev Phys Chem 58: 35–55.

[9] de Berg M, van Kreveld M, Overmars M, Schwarzkopf O (2000) Computational Geometry: Algorithms and Applications. Springer, second edition.
